# Evolving trends in lung cancer risk factors in the ten most populous countries: an analysis of data from the 2019 Global Burden of Disease Study

**DOI:** 10.1016/j.eclinm.2024.103033

**Published:** 2025-01-09

**Authors:** Chinmay T. Jani, Samuel A. Kareff, Dan Morgenstern-Kaplan, Ana S. Salazar, Georgina Hanbury, Justin D. Salciccioli, Dominic C. Marshall, Joseph Shalhoub, Harpreet Singh, Estelamari Rodriguez, Gilberto Lopes

**Affiliations:** a Sylvester Comprehensive Cancer Center at the University of Miami, Miami, FL, USA; b Jackson Health System, Miami, FL, USA; c Medical Data Research Collaborative, London, UK; d Lynn Cancer Institute, Baptist Health, Boca Raton, FL; e Department of Oncology, Guy's and St Thomas' Hospital, London, UK; f Division of Pulmonary and Critical Care, Brigham and Women's Hospital, Boston, MA, USA; g Harvard Medical School, Boston, MA, USA; h Imperial College Healthcare NHS Trust, London, UK; i Department of Surgery and Cancer, Imperial College London, London, UK; j Division of Interventional Pulmonology, Department of Pulmonary and Critical Care, University of California San Francisco

**Keywords:** Lung cancer, Air pollution, Disparities, Disease burden, Tobacco

## Abstract

**Background:**

Amid shifting tobacco policies and escalating air pollution levels, Lung Cancer (LC) risk factors have changed notably. Continuous assessment of these risk factors is necessary. This study compares trends in tobacco, air pollution, and asbestos-associated Age-Standardized Mortality Rates (ASMR) from Trachea, Bronchus, and Lung (TBL) Cancer across the top ten most populated countries (2023 censuses) and globally.

**Methods:**

We extracted overall and risk-factor-associated TBL cancer ASMR of the ten most populated countries for 1990–2019 from the Global Burden of Disease (GBD) database using the dedicated results tool (http://ghdx.healthdata.org/gbd-results-tool). GBD mapped the mortality data related to ICD codes (C33–C34, D02.1-D02.2, D38.1, 162–162.9, 231.1, 231.2, 231.8, 235.7 from ICD10 and B101 from ICD9). Tobacco, occupational exposure to asbestos and air pollution (ambient particulate matter and household air pollution) associated TBL cancer mortality data were extracted to evaluate the trends based on risk factors. We calculated relative changes in ASMRs between 1990 and 2019 for each sex in each country for each risk factor. Joinpoint regression analysis was performed to calculate the Estimated Annual Percentage Change (EAPC) and its corresponding 95% confidence interval (CI) for each line segment, allowing for trend assessment.

**Findings:**

Globally, TBL Cancer mortality has decreased by 8%, with a decrease for males but an increase for females. Globally, both tobacco and air pollution-associated TBL cancer ASMR have decreased. While tobacco-associated ASMR has increased in China and Indonesia, air pollution-associated ASMR has also increased in China, India, Pakistan, and Nigeria. On stratification, while PM-associated mortality increased by 25% globally, household-associated TBL cancer ASMR decreased by 62%. China had the highest PM-associated TBL Cancer in 2019 (8.8/100,000), twice higher than the global average. Despite a decline in asbestos-associated TBL cancer ASMR from 8.91/100,000 to 6.0/100,000, the rate in the United States remained twice higher than the global average for the entire study period.

**Interpretation:**

Tobacco-associated TBL cancer mortality is declining but still predominant. Ambient particulate matter-associated TBL cancer mortality is rising, requiring policy and awareness efforts. Expanding access to preventive services and addressing underlying risk factors are essential steps required toward reducing lung cancer mortality at the global level.

**Funding:**

This study did not receive any funding support.

**Research in context**

**Evidence before this study**

A PubMed search was conducted using terms such as “Lung cancer,” “Tracheal cancer,” “Bronchus cancer,” “Mortality,” “Trends,” and various risk factors, but no studies were found that comprehensively examined global TBL (Trachae Bronchus and Lung) cancer mortality trends while integrating risk factors. Upon narrowing the search to “Lung cancer,” “Tracheal cancer,” “Bronchus cancer,” “Mortality,” and “Trends,” we identified three relevant studies. One study projected trends in occupational lung cancer mortality, finding a global decline in mortality, although trends varied by country. Another study examining the effects of air pollution and greenspaces on TBL cancer mortality in the USA found positive associations with PM2.5 exposure and a potential protective effect of greenspaces. Narrowing the search to “Mortality,” “Risk factors,” and “Tracheal, bronchus, and lung cancer,” we identified studies that highlighted the trends in mortality and DALYs in the USA and China. Another study evaluating the Global, regional, and national burden of TBL cancer and its risk factors showed a decreasing trend in age-standardized incidence, mortality, and DALYs globally, with smoking as the major risk factor. While these studies offer insights into specific regions or risk factors, none have integrated a comprehensive range of risk factors, particularly asbestos and sub-granular air pollution data. Our study aims to fill this gap by analyzing global TBL cancer mortality trends, focusing on these risk factors.

**Added value of this study**

Our study provides the first comprehensive analysis of lung cancer mortality trends stratified by major risk factors—tobacco use, air pollution, and asbestos exposure—across the ten most populous countries, leveraging GBD data. By focusing on this specific set of countries, we delve deeper into the interplay of these risk factors, providing a detailed evaluation of their impact. Our findings highlight divergent trends, such as the global reduction in tobacco-associated mortality but rising mortality linked to ambient particulate matter. Using joinpoint regression analysis, we uncover disparities, such as the disproportionately high asbestos-associated mortality in the USA and the escalating particulate matter burden in China and India, while also incorporating a sex-specific lens to better understand how these risk factors differ by sex. These findings add valuable insights into global efforts to mitigate lung cancer mortality, emphasizing the need for tailored interventions based on country-specific and risk-specific profiles. This focused approach offers actionable data for addressing TBL cancer mortality in a significant portion of the global population.

**Implications of all the available evidence**

The findings underscore the need for continued global and regional efforts to control tobacco use, particularly among females, where trends are increasing in several countries. In addition, they emphasize the urgent need for stricter air quality policies to address the growing impact of particulate matter, especially in low- and middle-income countries. For asbestos, global bans and stronger regulatory frameworks are critical. These trends call for targeted public health initiatives, resource allocation, and further research into region-specific interventions, particularly in high-burden regions like China, India, and the USA.

## Introduction

Lung cancer (LC) stands as the foremost contributor to cancer-related mortality globally.^1^ In the United States of America (USA) alone, the National Cancer Institute's Surveillance, Epidemiology, and End Results (SEER) program projected 235,760 new cases and 131,880 deaths from lung cancer in 2021.^2^ Although advancements in targeted therapeutics have bolstered overall survival rates,^2^ significant variations persist across countries.^3^

Over recent decades, the landscape of LC risk factors has undergone a notable transformation.^3^ While tobacco smoking remains the primary culprit, there has been a rising prevalence of non-smoking-related LC.^4^ Compelling evidence suggests increased risk related to levels of air pollution,^5^ with a 29.3% surge in LC deaths attributed to ambient fine particulate matter (PM2.5) worldwide between 1990 and 2017.^6^ The synergistic impact of asbestos exposure and smoking has also been recognized in both the incidence and mortality rates of LC.^7^

Evolving trends in LC risk factors globally require ongoing reassessment of associated mortality. For instance, one study projected a global decline in occupational LC mortality, though trends varied by country, with some countries showing stable or increasing rates.^8^ Another study found positive associations between PM2.5 exposure and TBL cancer mortality in the USA, along with a potential protective effect of greenspaces.^9^ Study from the USA and China identified tobacco, air pollution, and diet low in fruits as key risk factors for TBL cancer in China, while tobacco, occupational carcinogens, and high fasting plasma glucose were significant in the USA.^10^ Additionally, a global burden study found a decrease in age-standardized incidence, mortality, and disability-adjusted life year (DALYs), with smoking as the major risk factor, but increases in DALYs due to secondhand smoke, high fasting plasma glucose, and occupational exposures, particularly in females while attributable age standardized mortality rate (ASMR) from ambient particulate matter pollution, household air pollution and low-fruit diets increasing in lower socio-demographic index (SDI) regions.^11^

While these studies provide valuable insights, none have integrated a comprehensive range of risk factors, particularly asbestos and granular air pollution data across multiple countries. This study compares trends in tobacco, air pollution and occupational asbestos-associated mortality from Trachea, Bronchus, and Lung (TBL) cancer across the top ten most populated countries and at the global level utilizing data from the Global Burden of Disease (GBD) database.

## Methods

This observational analysis of TBL cancer focuses on the ten most populous countries as determined by the most recent population estimates in 2023. Between 1990 and 2019, Japan, which was among the top ten most populous countries in 1990, was surpassed by other countries due to varying population growth rates. Conversely, Mexico entered the top ten during this period. By utilizing 2023 census data, our study captures the most current population dynamics, ensuring our findings are grounded in the latest demographic realities. Due to their substantial demographic weight, the ten most populous countries play pivotal roles in global affairs. They significantly influence global economic trends, environmental sustainability, and geopolitical stability. These countries collectively account for a large share of global resource consumption and greenhouse gas emissions, impacting international efforts toward risk factors affecting cancers.^12^^,^^13^

### Handling of the GBD data

We extracted ASMRs for TBL cancer globally and in the ten most populous countries from 1990 to 2019 using the dedicated GBD Results tool (http://ghdx.healthdata.org/gbd-results-tool).^14^ Age-standardized rates were applied to account for variations in the age structures of each country. Mortality data for tobacco use, air pollution, and occupational asbestos exposure associated with TBL cancer were extracted to assess trends based on these risk factors. To further explore air pollution trends, we obtained data for sub-categories, including ambient particulate matter and household pollution from solid waste, using the GBD database. We calculated the relative changes in ASMRs for each risk factor, by sex and country, between 1990 and 2019. The GBD methodology involves using a standard population based on the United Nations Population Division's World Population Prospects (2012 revision).^15^^,^^16^

### Characteristics of the data source

The GBD database consolidates datasets and registries from various countries, offering epidemiological insights into some of the world's most critical health issues. We have previously used similar methods to analyze trends in mortality from different cancers, including LC in the USA.^15^^,^^16^ The datasets employed by GBD researchers include insurance data, admission and outpatient encounter data, as well as systematic reviews, among others. Mortality and incidence data are mapped according to International Classification of Diseases (ICD) codes, including C33–C34, D02.1-D02.2, D38.1, 162–162.9, 231.1, 231.2, 231.8, and 235.7 from ICD10, and B101 from ICD9. These data are then combined using Bayesian meta-regression with the DisMod-MR 2.19 tool, which adjusts for bias and generates disease estimates with confidence intervals (CI).^14^^,^^17^ Since GBD 2010, the World Cancer Research Fund criteria have been used to establish convincing or probable evidence of risk–outcome associations, with systematic reviews updated for 81 pairs in GBD 2019. To assess exposure distributions for each risk factor by age, sex, location, and year, GBD utilized a range of sources, including published studies, household surveys, censuses, administrative data, ground monitoring data, and remote sensing data. For many risk factors, exposure data were modeled using either spatiotemporal Gaussian process regression or DisMod-MR 2.1, both Bayesian statistical models developed for GBD analyses. Further details on the data sources for each of these risk factors have been added in the methodology below. Additional details on the methodology are available on the GBD website and in previous publications.^15^^,^^16^^,^^18^^,^^19^

### Tobacco-associated mortality trends

The analysis included data on tobacco smoking derived from GBD's spatiotemporal Gaussian process regression (ST-GPR) framework, which estimates the prevalence of current and former smoking using nationally representative household surveys. Smoking prevalence was defined based on the use of any smoked tobacco product (daily or occasional).^20^ Relative risks for smoking-related outcomes, including LC, were calculated using integrated dose-response relationships, reflecting the cumulative effects of cigarettes per smoker per day and pack-years. These relationships were synthesized from cohort and case–control studies, adjusted for major confounders such as age and sex, and aligned with established methodologies from prior GBD cycles. For chewing tobacco, data on smokeless tobacco prevalence were incorporated using similar modeling strategies, emphasizing the inclusion of culturally specific products (e.g., betel quid with tobacco). This ensured that non-smoking tobacco exposure, a significant risk factor in some regions, was adequately captured. Population-attributable fractions (PAFs) were calculated for each risk factor using the prevalence of exposure and the corresponding relative risks. The PAFs for smoking, chewing tobacco, and secondhand smoke were assessed to estimate their contribution to the trends in TBL cancer mortality throughout the study period.^19^

### Air pollution-associated mortality trends

The analysis incorporated data on ambient particulate matter (PM2.5) and household air pollution (HAP) from the GBD database. Ambient PM2.5 exposure was defined as the population-weighted annual average concentration of particles ≤2.5 μm in diameter (μg/m³). Data sources included satellite observations (Hammer and colleagues Version V4.GL.03.NoGWR), ground measurements, chemical transport model simulations, and high-resolution population estimates.^21^^,^^22^ These inputs were integrated using the Data Integration Model for Air Quality 2 (DIMAQ2), which combines geospatial data with temporal trends to estimate PM2.5 concentrations for each region.^23^^,^^24^ Exposure levels were calibrated to ground monitoring data using spatially varying random effects to ensure alignment with measured concentrations. HAP exposure was estimated based on the proportion of individuals using solid cooking fuels (coal, wood, charcoal, dung) and associated PM2.5 concentrations, using data from standard multi-country surveys such as Demographic Health Surveys, Multiple Indicator Cluster Surveys and WHO Energy Database.^25^^,^^26^ A crosswalk adjustment was applied to translate household-level data to individual exposure. PM2.5 levels from HAP were modeled using sociodemographic variables like urbanization and maternal education and calibrated against field measurements. Risk exposure was mapped across regions using Gaussian process regression. For both ambient and household PM2.5 exposure, risk functions were derived using integrated dose-response relationships. GBD 2019 utilized flexible MR-BRT meta-regression to model relative risks, allowing for nuanced risk assessment across exposure levels. Theoretical minimum-risk exposure levels were defined as a uniform distribution between 2.4 and 5.9 μg/m³, reflecting the lowest observed risk levels in epidemiological studies. PAFs were calculated jointly for ambient and household air pollution to estimate their combined contributions to TBL cancer mortality.^19^

### Asbestos-mortality trends

Occupational asbestos exposure was estimated as the proportion of the population (aged 15 and older) exposed to asbestos during their lifetime. The asbestos impact ratio (AIR), derived from mesothelioma mortality rates, was used to calculate PAFs for asbestos-related diseases, excluding mesothelioma. The AIR was calculated using mesothelioma mortality data, incorporating differences in mortality rates among high-exposure, low-exposure, and unexposed populations. Primary inputs were sourced from GBD 2019 cause of death estimates and published studies, as well as data from the International Labour Organization (ILO), which provided information on employment across economic activities, fatal injury rates, and occupational distributions.^27^, ^28^, ^29^ Where data were unavailable, uncertainty estimates were generated using Loess curve fitting. ST-GPR was applied to estimate asbestos exposure prevalence across geographies and time periods. The ST-GPR models included random effects for regions and super-regions, with SDI and urbanization as covariates. The theoretical minimum risk level for asbestos exposure was assumed to be zero exposure. Relative risks for asbestos-associated diseases were obtained from systematic reviews and meta-analyses. These were last updated for GBD 2016 and included risks for cancers and other respiratory diseases.^19^

### Statistical analysis

Joinpoint regression analysis (using Joinpoint Command Line Version 4.5.0.1, USA National Cancer Institute Surveillance Research Program) was employed to analyze trends in mortality over time. This method allows us to model changes in the data by fitting a series of straight-line segments, each representing a different phase of the trend, on a logarithmic scale. The simplest model assumes a constant trend with no significant changes, while additional joinpoints represent points at which the trend significantly shifts. To determine where these shifts occur, the software uses a Monte Carlo permutation test to evaluate the statistical significance of each potential joinpoint. The goal is to ensure that only true, meaningful changes in the trend are included, while minimizing the risk of overfitting the model to noise in the data. In our analysis, we set the minimum number of joinpoints to 0 (i.e., no change in the trend) and the maximum to 3, which allows the model to capture up to three significant changes in the trend. This restriction ensures that the model remains parsimonious, avoiding unnecessary complexity.^16^

In our model, the Monte Carlo permutation test was conducted with 4499 permutations to rigorously assess the significance of each joinpoint. In this procedure, each hypothesis test compared the current model (with one additional joinpoint) to a simpler model (with fewer joinpoints). The null hypothesis that no significant change exists is rejected only when the inclusion of an additional joinpoint significantly improves the model fit, as indicated by a p-value less than 0.05. For each identified trend segment, the software calculates the Estimated Annual Percentage Change (EAPC) and its 95% CI. The EAPC quantifies the average annual change in the trend over that segment and provides a clear measure of the direction (upward or downward) and magnitude of change. A positive EAPC estimate, with the 95% CI entirely greater than 0, indicates an upward trend, while a negative EAPC, with the 95% CI entirely below 0, indicates a downward trend. This approach allows us to identify meaningful shifts in mortality trends, providing a nuanced understanding of how mortality has changed over time in relation to risk factors and other variables.

### Ethics

This study was conducted using an open-source database that includes anonymized patient information. Since the data utilized in the analysis does not contain any confidential or personally identifiable patient information, ethics approval was not required.

### Role of funding source

This study did not receive any funding support.

## Results

### Trends in overall TBL cancer mortality

Globally, the overall ASMR for TBL cancer decreased from 27.3/100,000 (1990) to 25.2/100,000 (2019), with an associated decrease in males (45.1/100,000–37.4/100,000) but an increase in females (13.0/100,000–15.0/100,000). In 1990, the overall ASMR was the highest in the USA (49.4/100,000), whereas in 2019, it was highest in China (38.7/100,000). ASMR increased in five countries (India, China, Indonesia, Pakistan, and Nigeria) and decreased in the remaining five countries (USA, Brazil, Bangladesh, Russia, and Mexico). The greatest change in overall ASMR was in the USA, with a reduction of 30%. Among males, in 1990, ASMR was the highest in Russia (76.1/100,000), followed by the USA (73.9/100,000), which by 2019 was found to be the highest in China (58.1/100,000). The greatest change in mortality rates in males was in the USA, with a reduction of 40% (73.9/100,000 to 44.2/100,000). Among females, ASMR in TBL cancer was by far the highest in the USA in both 1990 (31.5/100,000) and 2019 (29.6/100,000) (Table 1).

**Table 1:** Comparison of risk factor-associated TBL cancer ASMR (1990 vs 2019). A. Overall. B. Males. C. Females.

	Overall TBL ASMR N (1990 -->2019)	1990 Tobacco associated ASMR N (%)	2019 Tobacco-associated ASMR N (%)	1990 Air-pollution associated ASMR N (%)	2019 Air-pollution associated ASMR N (%)	1990 Asbestos associated ASMR N (%)	2019 asbestos associated ASMR N (%)	1990 Ambient particular matter associated ASMR N (%)	2019 Ambient particulate matter associated ASMR N (%)	1990 Household air pollution associated ASMR N (%)	2019 Household air pollution associated ASMR N (%)
**Overall**											
Global	27.3–>25.2	19.8 (72)	16.7 (66)	5.6 (20)	4.8 (19)	3.4 (12)	2.5 (10)	3.0 (11)	3.8 (15)	2.5 (9)	1.0 (4)
India	7.0–>8.1	4.2 (60)	4.0 (50)	2.5 (35)	2.6 (33)	0.3 (5)	0.5 (6)	0.7 (9)	1.7 (21)	1.8 (26)	0.8 (10)
China	31.2–>38.7	20.2 (65)	26.3 (68)	10.3 (33)	10.7 (28)	0.8 (2)	1.3 (3)	3.4 (11)	8.8 (23)	6.9 (22)	1.9 (5)
USA	49.4–>36.1	40 (81)	26.1 (72)	4.5 (9)	1.5 (4)	8.9 (18)	6.0 (17)	4.5 (9)	1.4 (4)	0.0 (0)	0.0 (0)
Indonesia	18.0–>24.4	10.6 (59)	14.7 (60)	5.4 (30)	4.6 (19)	0.2 (1)	0.9 (3)	1.3 (7)	2.8 (11)	4.0 (22)	1.8 (8)
Pakistan	14.7–>17.2	10.9 (74)	10.7 (63)	5.4 (36)	**5.5** (32)	0.3 (2)	0.6 (4)	0.9 (6)	3.0 (18)	4.4 (30)	2.4 (14)
Nigeria	6.8–>8.3	2 (29)	1.9 (23)	2.6 (38)	**2.7** (32)	0.2 (2)	0.1 (2)	0.4 (6)	1.3 (16)	2.2 (32)	1.3 (16)
Brazil	18.0–>15.8	13.9 (77)	9.7 (61)	3.2 (18)	1.5 (10)	1.5 (8)	1.3 (8)	1.3 (7)	1.2 (8)	1.9 (10)	0.3 (2)
Bangladesh	9.7–>7.8	6.9 (71)	4.7 (59)	3.6 (37)	2.6 (33)	0.5 (5)	0.2 (3)	0.5 (5)	1.2 (16)	3.1 (33)	1.4 (18)
Russia	32.4–>22.8	23.2 (72)	16.6 (73)	4.4 (13)	1.8 (8)	1.4 (4)	1.4 (6)	4.0 (12)	1.7 (8)	0.4 (1)	0.1 (0)
Mexico	15.1–>9.7	9.6 (63)	4.4 (45)	3.4 (22)	1.5 (16)	0.9 (6)	0.8 (8)	2.1 (14)	1.2 (13)	1.3 (8)	0.3 (3)
**Male**											
Global	45.1–>37.4	36.4 (81)	28.8 (77)	9.1 (20)	7.1 (19)	7.3 (16)	5.1 (14)	5.2 (11)	5.78 (15)	3.91 (9)	1.3 (4)
India	11.1–>11.7	7.6 (69)	7.3 (62)	3.9 (35)	3.7 (31)	0.6 (5)	1.0 (8)	1.1 (10)	2.57 (22)	2.77 (25)	1.1 (10)
China	46.3–>58.1	37.8 (82)	48.3 (83)	15.2 (33)	15.9 (27)	1.3 (3)	2.5 (4)	5.4 (12)	13.36 (23)	9.74 (21)	2.5 (4)
USA	73.9–>44.2	61.7 (83)	33.3 (75)	6.8 (9)	1.8 (4)	20.2 (27)	11.9 (27)	6.7 (9)	1.76 (4)	0.02 (0)	0.0 (0)
Indonesia	17.4–>35.8	20.6 (75)	28.0 (78)	8.1 (30)	6.7 (19)	0.4 (2)	1.8 (5)	2.2 (8)	4.22 (12)	5.95 (22)	2.4 (7)
Pakistan	24.1–>27.9	18.9 (78)	19.5 (70)	8.8 (36)	8.9 (32)	0.5 (2)	1.1 (4)	1.6 (6)	5.06 (18)	7.19 (30)	3.8 (14)
Nigeria	11.4–>12.1	3.9 (34)	3.6 (30)	4.3 (38)	3.9 (33)	0.3 (3)	0.2 (2)	0.8 (7)	1.98 (16)	3.58 (31)	1.9 (16)
Brazil	18.1–>20.9	22.9 (81)	14.2 (68)	5.0 (18)	2.0 (10)	2.6 (9)	2.3 (11)	2.2 (8)	1.63 (8)	2.78 (10)	0.4 (2)
Bangladesh	15.3–>11.6	12.0 (78)	8.3 (72)	5.7 (37)	3.9 (33)	0.8 (5)	0.4 (3)	0.8 (5)	1.89 (16)	4.94 (32)	2.0 (17)
Russia	76.1–>47.4	62.2 (82)	39.4 (83)	10.2 (13)	3.7 (8)	3.8 (5)	3.6 (7)	9.4 (12)	3.63 (8)	0.86 (1)	0.1 (0)
Mexico	21.6–>13.8	15.8 (73)	7.7 (56)	4.8 (22)	2.2 (16)	1.6 (7)	1.5 (11)	3.1 (14)	1.77 (13)	1.71 (8)	0.4 (3)
**Females**											
Global	13.0–>15.0	6.5 (49)	6.6 (44)	2.7 (21)	2.7 (18)	0.6 (4)	0.6 (4)	1.30 (10)	2.1 (14)	1.4 (11)	0.65 (4)
India	2.9–>4.7	0.7 (23)	1.0 (21)	1.0 (36)	1.5 (32)	0.1 (2)	0.1 (2)	0.21 (8)	0.9 (20)	0.8 (28)	0.57 (12)
China	18.6–>22.9	5.5 (30)	8.0 (35)	6.3 (34)	6.4 (28)	0.4 (2)	0.5 (2)	1.69 (9)	5.0 (22)	4.6 (24)	1.39 (6)
USA	31.5–>29.6	24.1 (77)	20.2 (68)	2.9 (9)	1.2 (4)	1.4 (4)	1.5 (5)	2.86 (9)	1.2 (4)	0.01 (0)	0.00 (0)
Indonesia	9.7–>14.7	1.8 (19)	3.5 (23)	2.9 (30)	2.9 (20)	0.1 (1)	0.2 (1)	0.60 (6)	1.6 (11)	2.3 (24)	1.33 (9)
Pakistan	3.1–>5.8	1.0 (32)	1.4 (25)	1.2 (38)	1.9 (32)	0.1 (2)	0.1 (2)	0.14 (5)	0.9 (15)	1.0 (33)	0.96 (17)
Nigeria	2.7–>4.7	0.3 (11)	0.4 (7)	1.0 (38)	1.5 (32)	0.04 (2)	0.1 (1)	0.15 (6)	0.8 (16)	0.9 (32)	0.74 (16)
Brazil	9.4–>11.8	6.3 (67)	6.3 (53)	1.7 (18)	1.2 (10)	0.6 (6)	0.7 (6)	0.62 (7)	0.9 (8)	1.1 (12)	0.29 (2)
Bangladesh	2.6–>3.6	0.6 (21)	0.6 (16)	1.0 (39)	1.2 (34)	0.1 (2)	0.1 (2)	0.09 (4)	0.5 (13)	0.9 (35)	0.76 (21)
Russia	8.5–>7.7	2.3 (27)	2.8 (36)	1.2 (14)	0.6 (8)	0.3 (3)	0.3 (3)	1.02 (12)	0.6 (8)	0.1 (2)	0.02 (0)
Mexico	9.1–>6.3	3.8 (41)	1.5 (24)	2.1 (23)	1.0 (16)	0.3 (3)	0.2 (3)	1.20 (13)	0.8 (13)	0.9 (10)	0.22 (3)

ASMR, Age Standardized Mortality Rate; TBL, Trachea Bronchus and Lung Cancer; USA, United States of America. N is presented as per 100,000 population. % represents the proportional ASMR due to each risk factor highlighted in specific columns.

Upon stratification by risk factors, including environmental, behavioral, and metabolic risks, the TBL cancer ASMR associated with any identified risk factors in GBD 2019 decreased from 84% to 81%, with a corresponding reduction from 90% to 88% in males and from 70% to 65% in females.

### Trends in tobacco-associated TBL cancer mortality

**Fig. 1:**
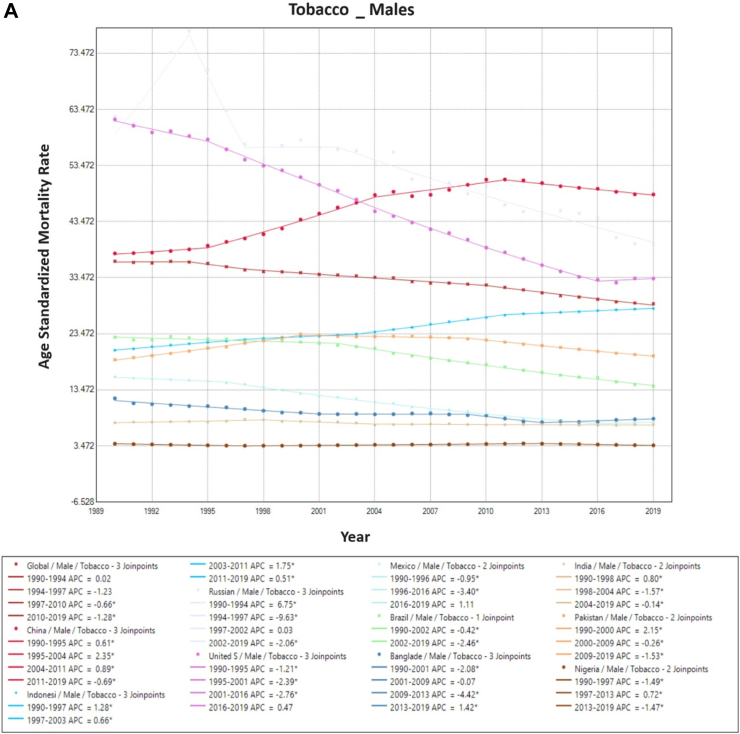

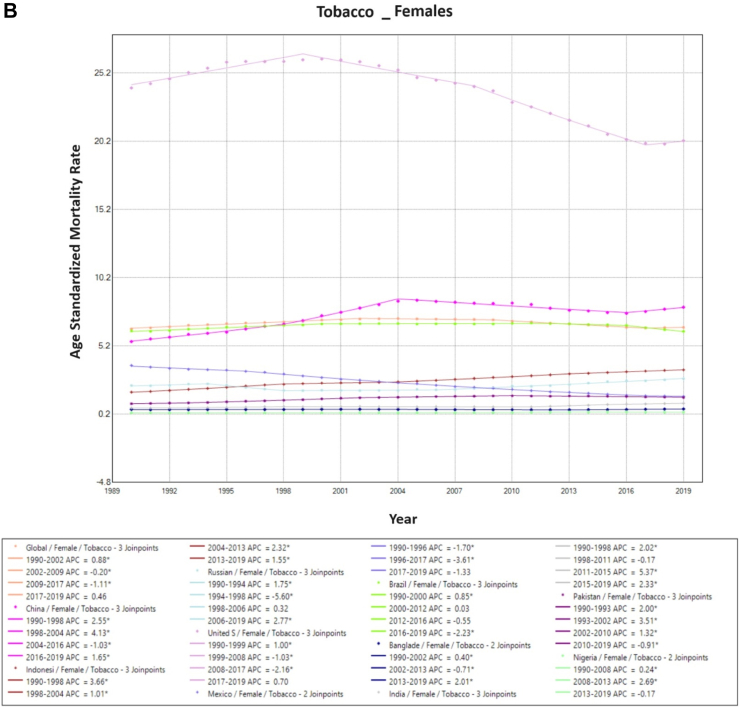
**Age-Standardized Mortality Rates (ASMR) trends for Tobacco. All indices are per 100,000 population**. ASMR, Age Standardized Mortality Rate; APC, Annual Percentage Change.

The proportion of tobacco-associated ASMR in TBL cancer decreased worldwide from 72% to 66%. The proportional mortality rate from tobacco-associated TBL cancer fell in both males (81%–77%) and females (49%–44%). Tobacco-associated ASMR rose in China (20.2/100,000–26.3/100,000) and Indonesia (10.6/100,000–14.7/100,000) from 1990 to 2019, with a reduction in mortality in the remaining countries. Among males, tobacco-associated ASMR fell over time across all countries, excluding China, Indonesia, and Pakistan. Among females, the reverse pattern was observed with overall increasing rates of tobacco-associated ASMR in most countries, with only rates in the USA and Mexico falling between 1990 and 2019 (Fig. 1).

On Joinpoint analysis, ASMR has decreased globally since 1994, with males having a constant decline since 1990, while females had a decreasing trend since 2012, then a change with an increasing trend since 2017. On evaluating trends in each country for males, most of the countries have shown a decline in recent years except the USA (EAPC 0.5%, 2016–2019), Mexico (EAPC 1.1%, 2016–2019), Bangladesh (EAPC 1.4%, 2013–2019), and Indonesia (EAPC 0.5%, 2011–2019). For females, there has been an increasing ASMR in recent years in 6 of the 10 countries (China, Indonesia, Russia, USA, Bangladesh, and India), with the highest increase observed in Russia (EAPC 2.8% since 2006). Furthermore, the decline in tobacco-associated TBL cancer ASMR was noted to occur later in females compared to males. This trend was observed globally and across all countries except Mexico at the onset of the study period, with most countries experiencing an increase in the first decade (1990s) for females. Conversely, for males only China, India, Pakistan, and Indonesia observed an increase in ASMR in the first decade (Supplementary Tables S1–S3).

### Trends in occupational exposure to asbestos-associated TBL cancer mortality

**Fig. 2:**
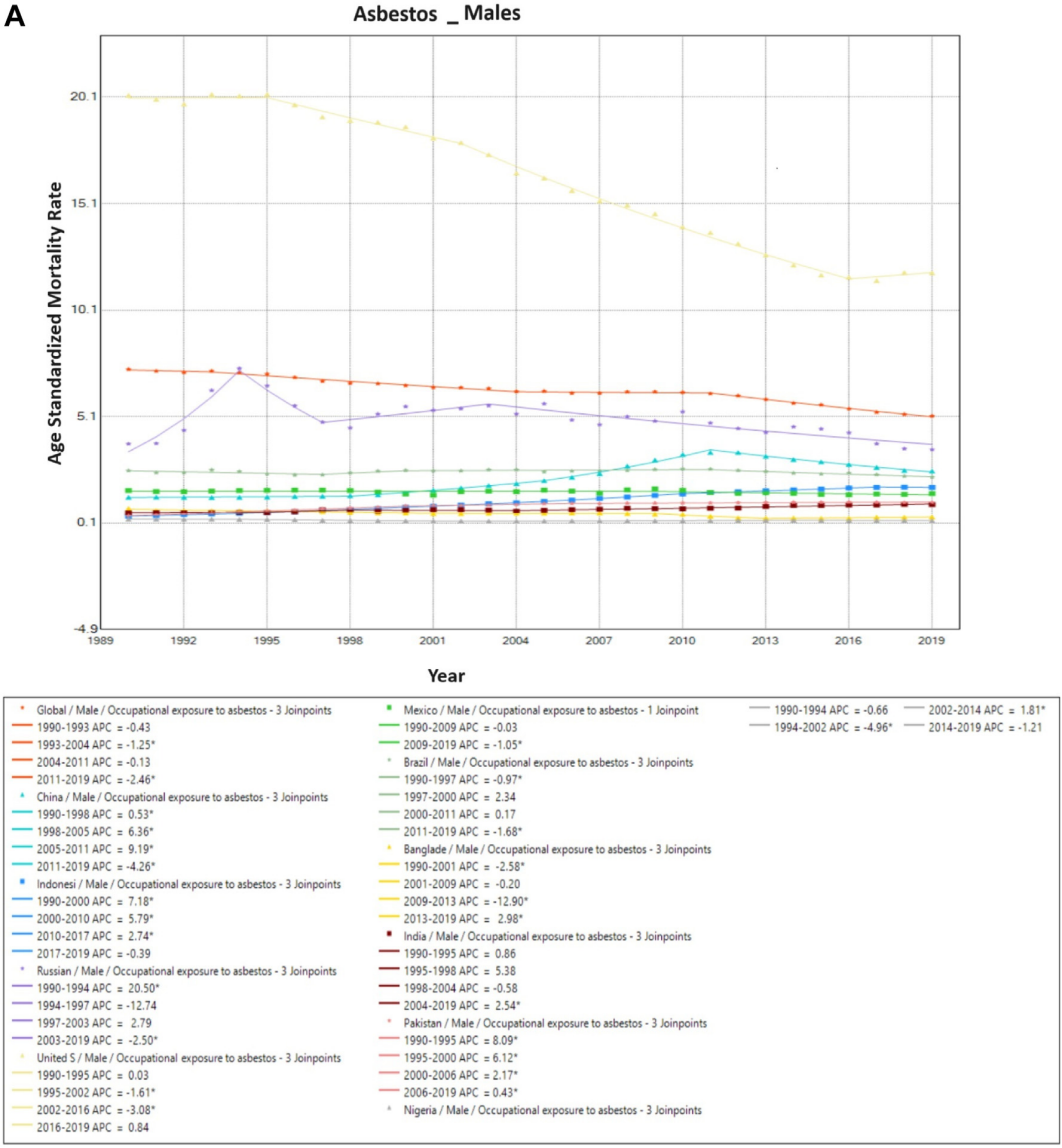

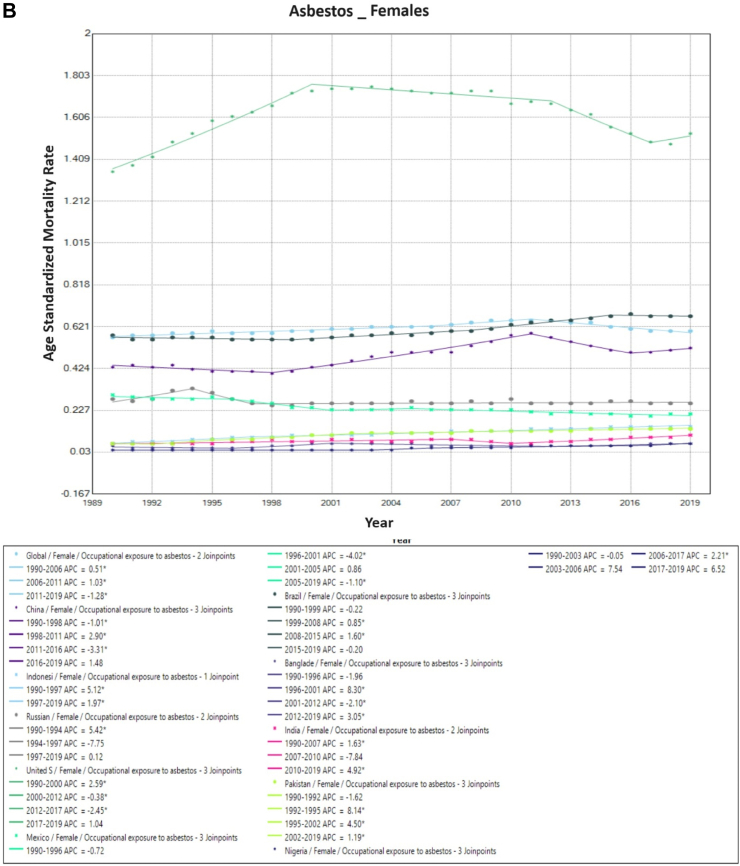
**Age-Standardized Mortality Rates (ASMR) trends for Occupational Exposure to Asbestos. All indices are per 100,000 population.** ASMR, Age Standardized Mortality Rate; APC, Annual Percentage Change.

Global asbestos-associated TBL cancer ASMR has fallen from 3.4/100,000 (1990) to 2.5/100,000 (2019). This is primarily due to a fall in ASMR among males (7.3/100,000–5.1/100,000) as the rates among females remained constant (0.6/100,000 in 1990 and 2019). Among males, even though asbestos-associated TBL cancer rates have fallen by 50% in the USA, they have remained by far the highest throughout the study period (1990: 20.2/100,000; 2019: 11.9/100,000). Despite this decrease in ASMR in males in the USA, rates remained twice as high as the global average for both males and females throughout the study period. In males, there was a fall in asbestos-associated mortality rates in 6 of the 10 countries, with an increase seen only in India, China, Indonesia, and Pakistan. Among females, a marginal reduction in ASMR was observed in Mexico (0.3/100,000 in 1990–0.2/100,000 in 2019), however rates were maintained or increased over time in all remaining countries (Fig. 2).

On Joinpoint analysis, globally, there has been a decline for males throughout the study period, with females showing a decline in recent years since 2011. In recent years, for males, six countries showed a decline except the USA (EAPC 0.8%, 2016–2019), Pakistan (EAPC 0.4%, 2006–2019), India (EAPC 2.6%, 2004–2019) and Bangladesh (EAPC 3.0%, 2013–2019). Conversely, for females, all countries have shown an increase in recent years except Mexico and Brazil, with the highest increase observed in India (EAPC 4.9%, 2010–2019) (Supplementary Tables S1–S3).

### Trends in air-pollution-associated TBL cancer mortality

**Fig. 3:**
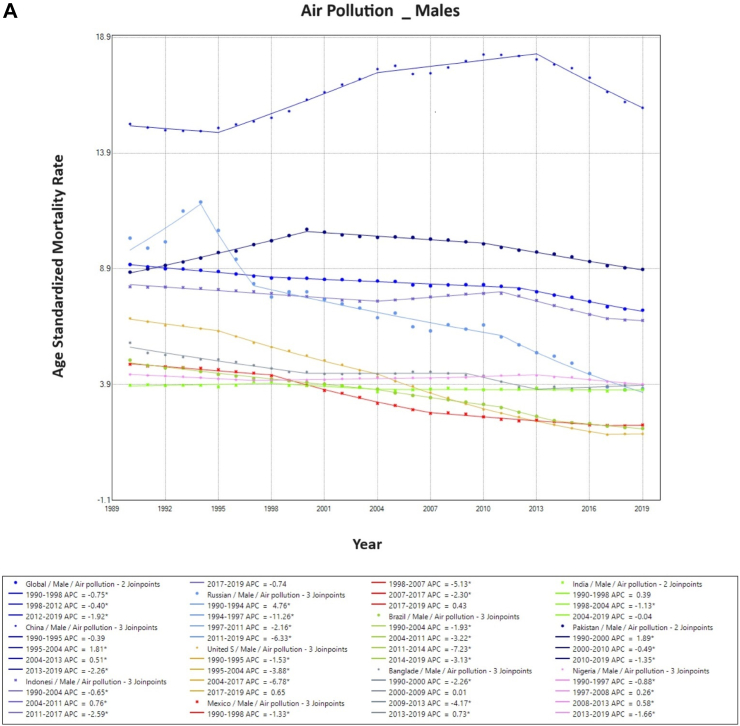

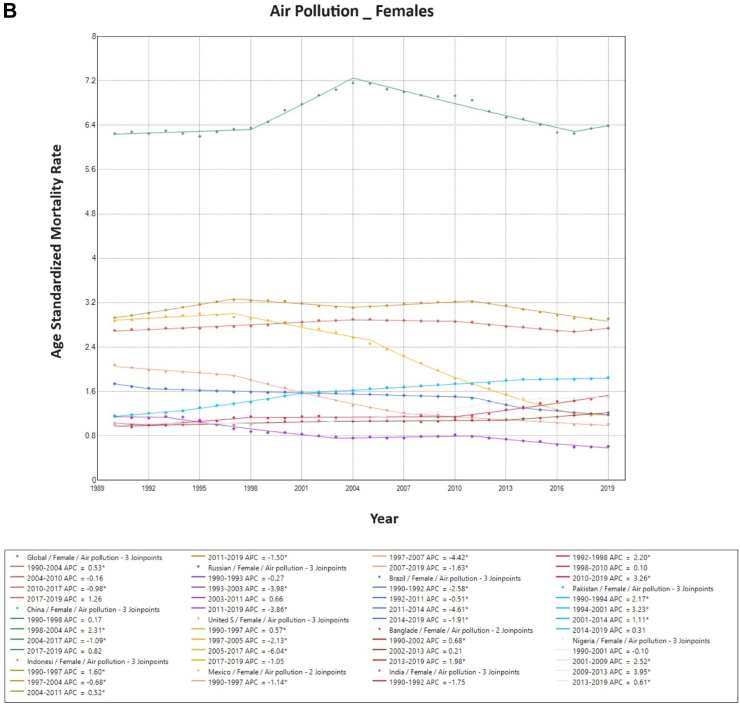
**Age-Standardized Mortality Rates (ASMR) trends for air pollution. All indices are per 100,000 population**. APC, Annual Percentage Change; ASMR, Age Standardized Mortality Rate.

Air pollution-associated TBL cancer ASMR has reduced globally during the observation period. This is reflected in both sexes, with declining rates across all countries except China and Pakistan for males, and India, China, Pakistan, Nigeria and Bangladesh for females. Overall, China showed a significantly higher ASMR than the other nine countries for both males and females. With an increase in ASMR in 2019, China had almost double the ASMR as compared to the global level for males and females (Fig. 3).

On Joinpoint analysis, ASMR has decreased globally since 1994, with males having a constant decline since 1990 while females have been showing a decline since 2004 with a concerning increase from 2017 onwards. On evaluating trends in each country, for males, most of the countries have shown a decline in recent years except the USA (EAPC 0.7%, 2017–2019), Mexico (EAPC 0.4%, 2017–2019), and Bangladesh (EAPC 0.7%, 2013–2019). Whereas in females, five countries, including China, Bangladesh, India, Pakistan, and Nigeria, have shown an increase in ASMR in recent years, with the highest ASMR change observed in recent years in India (EAPC 3.3%, 2010–2019) (Supplementary Tables S1–S3).

### Sub-group analysis: trends in ambient particulate matter and household air pollution from solid fuels-associated TBL cancer mortality

Upon further stratifying air pollution trends, overall ambient particulate matter-associated mortality has increased globally. Among males, a marked increase in ASMR was observed in China (5.4/100,000–13.36/100,000 for males, and 1.69/100,000–5.0/100,000 for females.). Apart from China, among males, an increase was observed in five other countries (Pakistan, Indonesia, India, Nigeria and Bangladesh). Similarly, in females, an increase was observed in 7 of the 10 countries except Russia, the USA, and Mexico. Whereas, globally, TBL cancer secondary to household air pollution has been on the decline (2.5/100,000–1.0/100,000). This reduction in mortality was seen in males and females in each country (Table 1).

**Fig. 4:**
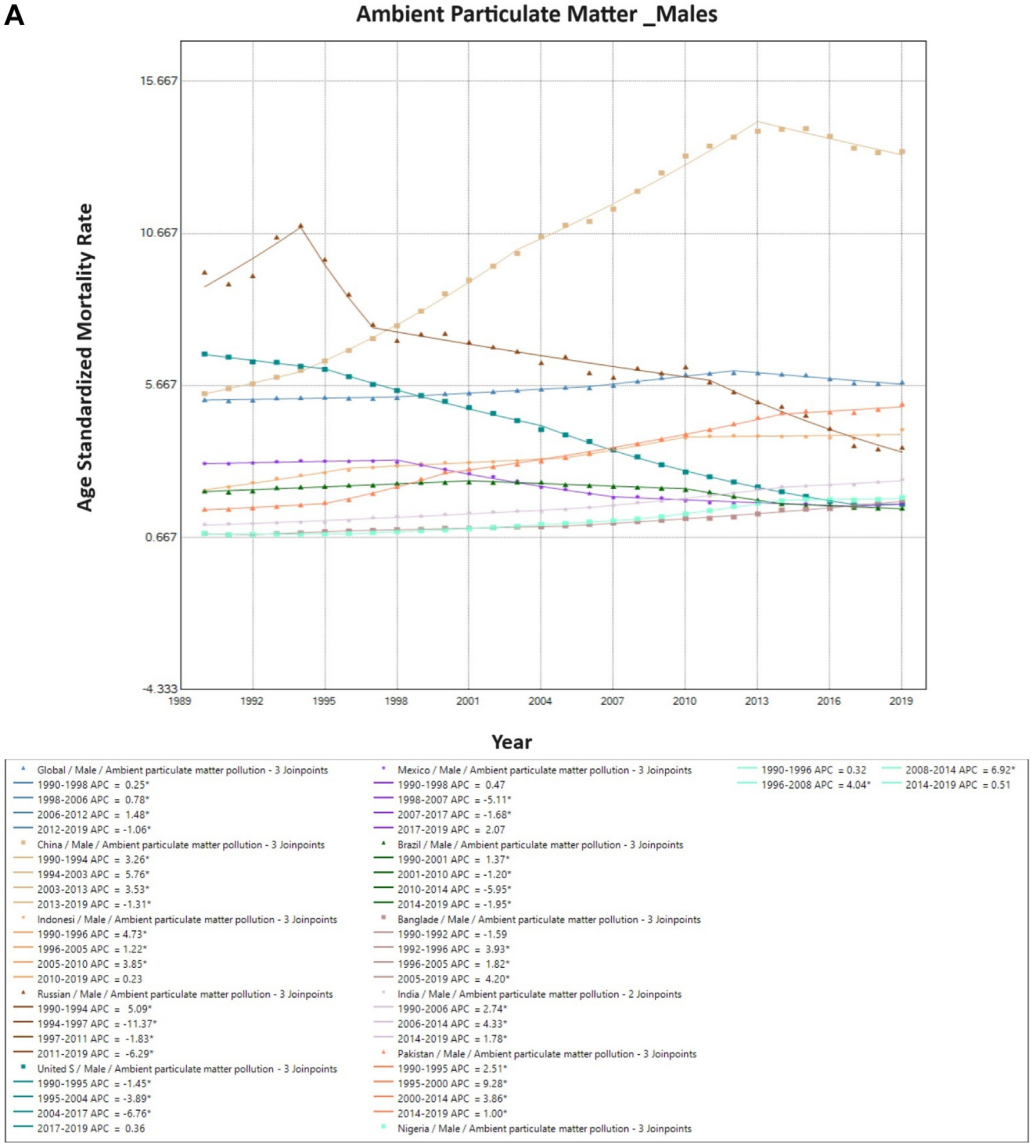

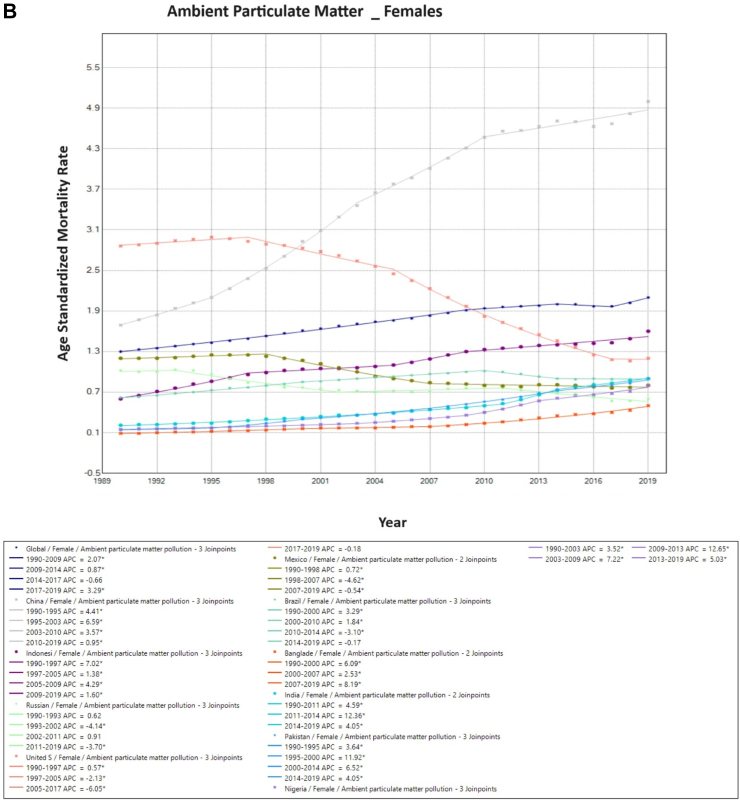
**Age-Standardized Mortality Rates (ASMR) trends for ambient particulate matter. All indices are per 100,000 population**. ASMR, Age Standardized Mortality Rate; APC, Annual Percentage Change.

**Fig. 5:**
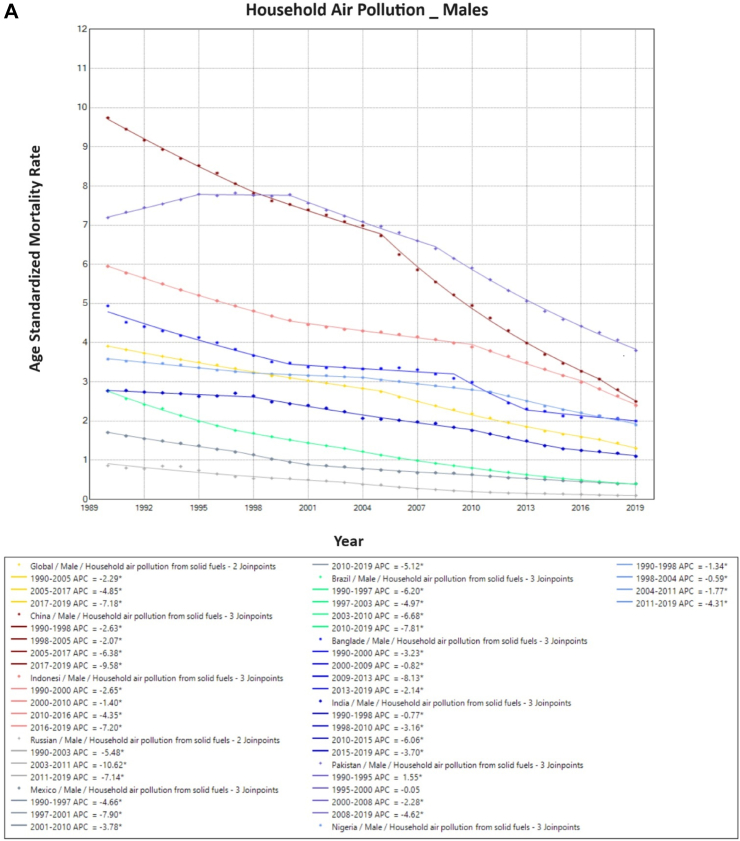

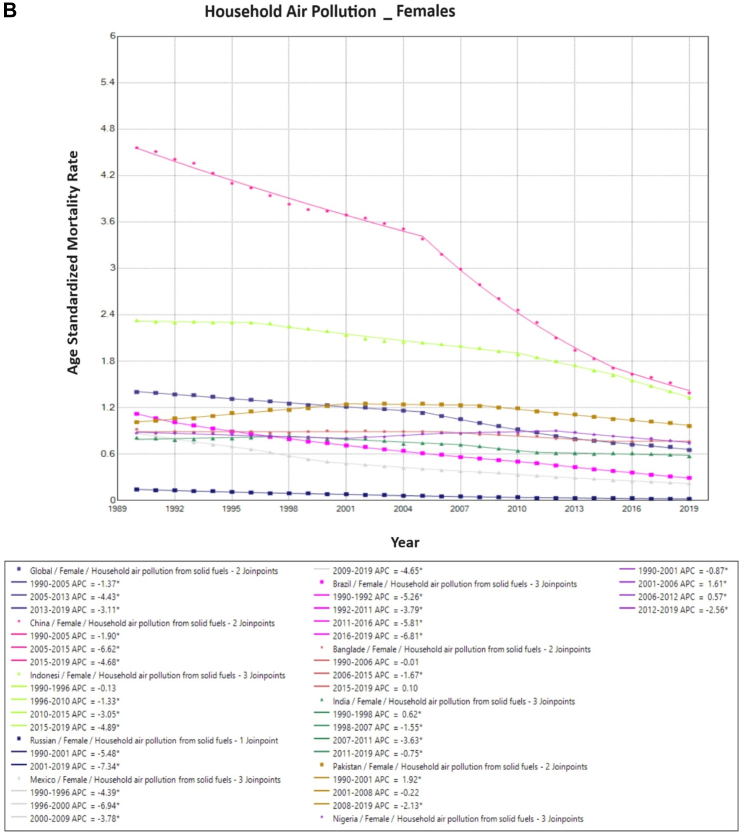
**Age-Standardized Mortality Rates (ASMR) trends for household air pollution. All indices are per 100,000 population**. ASMR, Age Standardized Mortality Rate; APC, Annual Percentage Change.

On Joinpoint analysis, ambient particulate associated TBL cancer ASMR globally in males increased constantly until 2012, with a recent decline of EAPC −1.1%. Conversely, for females, there has been a constant increase throughout the study period except for the time period of 2014–2017. On evaluating trends in each country, the ASMR was found to increase in the first decade for females globally (1990s), which was similar for males except in the USA (EAPC −1.5%, 1990–1995). Surprisingly, in recent years, there has been an increase observed globally as well as in 6 of the 10 countries for females (China, India, Pakistan, Bangladesh, Nigeria, and Indonesia), with the highest increase observed in Bangladesh (EAPC 8.2%, 2017–2019). Similarly, for males, 7 of the 10 countries have shown an increase in recent years except China, Brazil, and Russia, with the highest increase in Bangladesh (EAPC 4.2%, 2005–2019) (Fig. 4). For household pollution-associated TBL cancer ASMR, there has been a decrease in ASMR globally and in all countries for the entire study period except for males in Pakistan (EAPC 1.6%, 1990–1995), and for India (EAPC 0.6%, 1990–1998), Pakistan (EAPC 1.9%, 1990–2001) and Bangladesh (EAPC 0.1%, 2015–2019) for females (Supplementary Tables S1–S3, Supplementary Figure S1) (Fig. 5).

## Discussion

TBL cancer mortality trends have evolved over the past three decades.^3^ We observed that even though there was a reduction in overall TBL cancer mortality worldwide, ambient particulate matter-associated TBL cancer mortality has increased globally with a steady decline for tobacco-associated proportional TBL cancer mortality. Contrasting results were observed amongst the 10 most populous countries of the world, with China having the highest burden of disease in 2019 as compared to the USA in 1990. We also observed concerning trends related to different risk factors in specific countries with an ongoing increase in tobacco-associated mortality in females in 5 of the 10 countries studied, an ASMR double the global average for air pollution in China and asbestos-associated ASMR in the USA. To our knowledge, this paper represents the first attempt to compare trends in tobacco use, air pollution, and occupational asbestos-associated mortality related to TBL cancer across the ten most populated countries. In the following section, we will discuss each risk factor individually.

Tobacco remains the most contributory risk factor for LC mortality worldwide. In our study, we observed an overall decrease in tobacco-associated TBL cancer mortality globally. However, it still accounted for 66% of TBL cancer mortality in 2019. Global and national anti-tobacco policies and awareness have helped in reducing tobacco uptake, thereby reducing LC mortality.^30^^,^^31^ Epidemiological studies have confirmed a dose-response relationship with peaks in the smoking epidemic and LC mortality.^31^ Countries with early initiatives to reduce smoking prevalence have shown a steady decline in LC.^3^ For example, in 1990, the USA had the highest tobacco-associated TBL cancer ASMR of 40/100,000, almost double the global averages. However, there has been a significant decline throughout the study period, reflecting successful policy implementation and awareness leading to a reduction in smoking prevalence amongst its population.^32^ Furthermore, the proportion of heavy smokers (i.e., >30 cigarettes daily) has also significantly declined.^32^ Anti-smoking public health campaigns in the USA have both proved cost-effective and reduced smoking prevalence.^30^^,^^33^

However, contrasting trends were observed in China, with tobacco-associated TBL cancer ASMR increased to the highest amongst the ten countries. Surprisingly, there was also an increase in proportional TBL cancer ASMR due to tobacco from 65% to 68%. These have resulted from a steady increase in smoking prevalence, leading to a constant increase in LC mortality in China.^34^ Similarly, a proportional increase in tobacco-associated TBL cancer ASMR was observed in Indonesia. High smoking prevalence is observed in Indonesia, with the highest tobacco-attributable cancer incidence amongst all Southeast Asian males and females.^35^

Upon evaluating trends by sex, while there was a reduction in tobacco-associated TBL cancer mortality in 7 of the 10 countries for males, however, 6 of the 10 countries showed an increase for females. Overall, this was reflective of the global trends in tobacco consumption across these countries.^36^ It has also been observed that tobacco peaks have lagged in females, which leads to subsequent delays in peaks in LC mortality.^31^ Despite historically lower smoking prevalence among females, there has been a rise in many regions. While it has been found that females might be more susceptible to cigarette carcinogens,^3^ targeted tobacco marketing, greater accessibility, and lower prices may contribute to the rising female rates.^36^

Air pollution has now emerged as the second most common risk factor, accounting for nearly 20% of TBL cancer ASMR globally and more than 25% in China, India, Pakistan, Bangladesh, and Nigeria. On further stratification, overall ambient particulate matter-associated mortality has increased globally with a reduction in household air pollution.

Our study showed an alarming increase in ambient matter-associated TBL cancer ASMR in 7 of the 10 countries for males and 6 of the 10 countries for females, with a significantly higher ASMR in males compared to females. This could be due to an increase in the prevalence of PM2.5, which can have deeper lung penetration.^37^ Global estimates of PM2.5 concentrations have shown increasing trends globally (0.04 ± 0.02 μg/m^3^/yr).^21^ With increased exposure to higher concentrations of particulate matter, there is a causal and linear relationship between excess risk for LC, particularly with PM2.5 exposure.^21^^,^^37^

In our study, China in 2019 had the highest ASMR for ambient particulate matter-related TBL cancer. On Joinpoint analysis in our study, the highest EAPC in recent years was observed in India for males and in Bangladesh for females. Estimates for PM2.5 have also shown a similar alarming increase, especially in India over 2005–2013 (2.44 ± 0.44 μg/m^3^/yr).^21^ While tobacco campaigns have gained significant momentum worldwide, future campaigns and stricter policies are needed to target these pollutants.

Asbestos exposure has been associated with LC since the early 1900s, with an overall quantitatively greater association with primary LCs compared to pleural mesothelioma.^38^ With growing evidence of asbestos leading to LC,^38^ guidelines and policies have been formulated to reduce asbestos utilization.^39^ We observed a reduction in asbestos-associated TBL cancer (26%), with a major reduction in ASMR in males (30%). Despite this global decrease, alarming rates remained in the USA, with almost three times higher ASMR compared to the global average, especially in males. Concerning increases were also seen in India, China, Indonesia, and Pakistan. There remains significant exposure to asbestos in India, China, and many other developing countries, with an estimated 125 million people ever exposed as of 2018 and use of almost 2 million tons as of the recent past decade.^40^ Adoption of the C162 Asbestos Convention and the Basel Convention with facilitation for a total asbestos ban is the need of time at the global level.^41^ The recent ban by the USA Environment Protection Agency was much needed to reduce the impact of asbestos on chemical and health safety.^42^

Our study revealed a decrease in tobacco-associated TBL cancer ASMR from 72% in 1990 to 66% in 2019, with rates of approximately 77% for males and 44% for females. This shift reflects an increasing proportion of LC cases now occurring in never-smokers. Molecular profiling using next-generation sequencing has emerged as a critical diagnostic tool, with studies showing that 95% of never-smokers had a driver mutation, 78% of which were actionable, compared to 75% driver mutations and 47% actionable mutations in smokers.^43^

At the same time, the relationship between LC incidence and mortality is multifaceted, shaped by evolving risk factors, advancements in early detection, and therapeutic progress. One limitation of the GBD database is its inability to provide data linking incidence directly to specific risk factors. However, in one of our previous studies, the USA showed declining incidence and mortality rates, particularly among males, highlighting the success of reduced smoking prevalence and improved treatment options.^16^ However, the rising incidence in females in industrialized countries reflects changing smoking patterns, higher susceptibility to carcinogens, and delayed peaks in smoking trends.^11^^,^^44^ In contrast, emerging economies, such as India, show diverse smoking patterns and increasing LC incidence and mortality in both males and females, compounded by risks from widespread air pollution.^45^ This underscores the need for targeted interventions.^11^^,^^44^

Despite advancements in diagnostics and therapeutics, the increasing proportion of non-smoking as well as non-risk-factor-associated LC mortality rates underscores the critical need for comprehensive approaches to LC control and treatment. For instance, LC screening has shown a significant reduction in LC mortality.^3^ However, implementing it on a population-wide scale remains challenging. Screening is especially crucial among females, given the concerning trend of increasing tobacco-associated LC ASMR in females.^3^ Furthermore, with an increasing proportion of non-smoking-associated LC, modifications in LC screening guidelines are urgently needed. Risk prediction models derived from large-scale studies offer an opportunity to include additional risk factors and occupational exposures, such as asbestos, to better define eligible populations. However, their widespread implementation has only recently begun, requiring further refinement and adoption.^46^

Our trend analysis utilizing Joinpoint allows for the assessment of population-level trends over an extended observation period using the annual mortality data collected from the GBD. However, the GBD has inherent limitations that the GBD study collaborators have previously outlined.^3^^,^^16^ First, there is an alteration in the data coding system and country-specific practices over the study period, particularly the transition from ICD9 to ICD10. However, the GBD authors addressed this by mapping mortalities to the cause-of-death lists for coding system adjustments. The second limitation is the data heterogeneity and variability, which can arise from differences in data sources, regional disparities in exposure to risk factors such as tobacco use, air pollution, and occupational hazards, as well as temporal changes in socioeconomic and regulatory environments across countries. Additionally, variability in the reliability of death certification contributes to this heterogeneity, with global error rates ranging from 39% to 61%.^16^^,^^47^ Furthermore, the GBD uses garbage code distribution algorithms and corrections to label deaths resulting from poorly defined diagnoses or those that cannot scientifically be the sole underlying cause of death.^18^^,^^19^ Despite these challenges, the use of Joinpoint regression allowed us to analyze these trends effectively. By grouping results, it accounts for and navigates this inherent variability, underscoring its utility in managing the complexities of real-world data. The third limitation is the inability to subcategorize the individual histologic and staging subtypes of LC from the GBD as histopathologic subtypes and stages of LC have varying clinical significance and management. Finally, our study is observational and based on estimation, which should complement the available reported data. Further causal inferences apart from risk factors associated ASMR cannot be inferred. For systematic bias in risk exposure data, the GBD study aims to correct this by establishing a reference definition of each exposure and adjusting acceptable alternative measurements on the basis of studies with observed data pairs of the two different definitions. However, after these adjustments, residual measurement bias may persist and vary around the world.^19^

The fluctuating trends in tobacco-associated and the concerning rise in air pollution-associated TBL cancer mortality highlight the complex interplay of societal and environmental factors. While tobacco-associated TBL cancer mortality is declining globally, it remains the most significant factor, amounting to three-quarters of cases involving males. Continued efforts are necessary to control tobacco. Conversely, ambient particulate matter-associated TBL cancer mortality is increasing worldwide, demanding special attention for policy formulation and awareness campaigns, particularly in lower- and middle-income countries. Expanding access to preventive services and addressing underlying risk factors are essential steps required toward reducing lung cancer mortality at the global level.

## Contributors

Chinmay Jani: Conceptualization, data curation, formal analysis, methodology, writing original draft.

Samuel A. Kareff: Conceptualization, methodology, writing original draft, review and editing.

Dan Morgenstern: Methodology, writing-original draft.

Ana Salazar: Methodology, writing-original draft.

Georgina Hanbury: Writing-original draft.

Justin D Salciccioli: Data curation, methodology, review and editing.

Dominic C Marshall: Methodology, writing-review and editing.

Joseph Shalhoub: Methodology, writing-review and editing.

Harpreet Singh: Conceptualization, methodology, writing-original draft.

Estelamari Rodriguez: Methodology, Supervision, writing-review and editing.

Gilberto Lopes: Conceptualization, methodology, supervision, Writing–review and editing.

Chinmay Jani, Ana Salazar and Harpreet Singh have accessed and verified the data, and all authors were responsible for the decision to submit the manuscript. All authors read and approved the final version of the manuscript.

## Data sharing statement

Data used for the analyses are publicly available from the Institute of Health Metrics and Evaluation (http://www.healthdata.org/; http://ghdx.healthdata.org/gbd-results-tool).

## Declaration of interests

All authors declare no competing interests.
