# Supplementary material for: Evolving trends in lung cancer risk factors in the ten most populous countries: an analysis of data from the 2019 Global Burden of Disease Study
Source: eClinicalMedicine. 2025 Jan 9;79:103033. doi: 10.1016/j.eclinm.2024.103033 (PMC11833020; doi:10.1016/j.eclinm.2024.103033)
Supplement: Supplementary Figure [file mmc1.docx]

**Supplementary Figure 1: Overall Age-Standardized Mortality Rates (ASMR) trends for TBL cancer based on association with risk factors. All indices are per 100,000 population.**

(A)


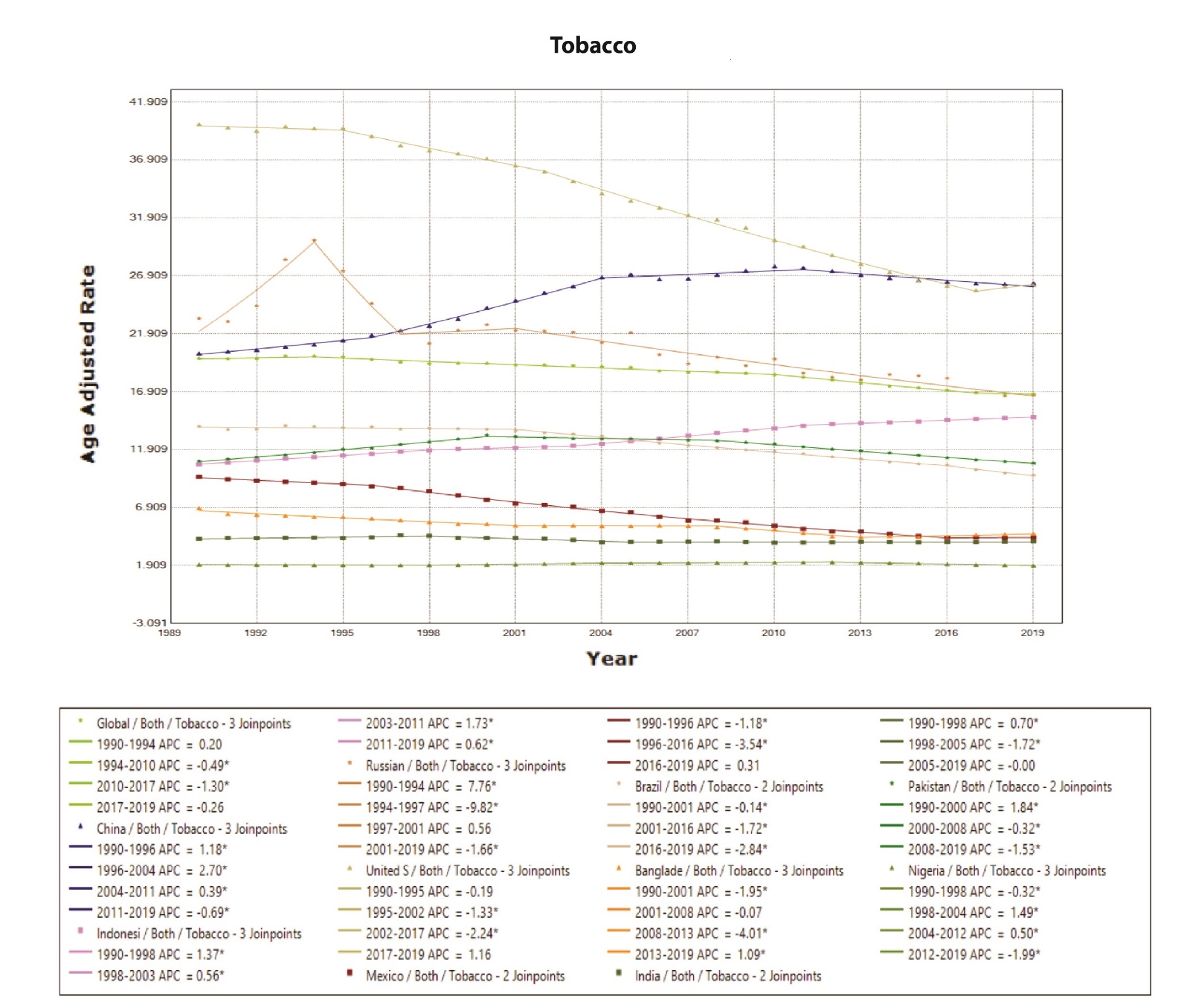


ASMR: Age Standardized Mortality Rate; APC: Annual Percentage Change

(B)


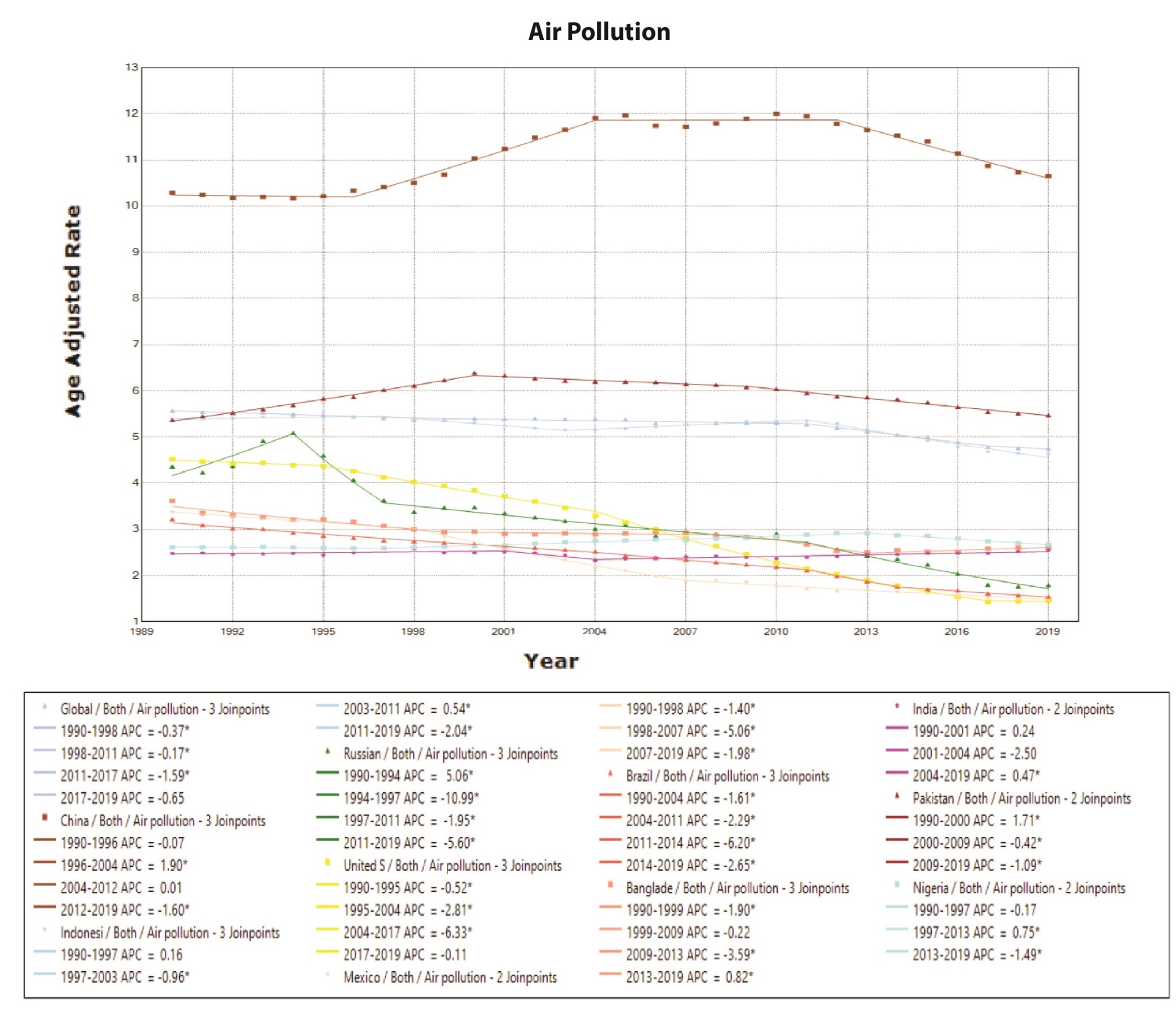


ASMR: Age Standardized Mortality Rate; APC: Annual Percentage Change

( C )


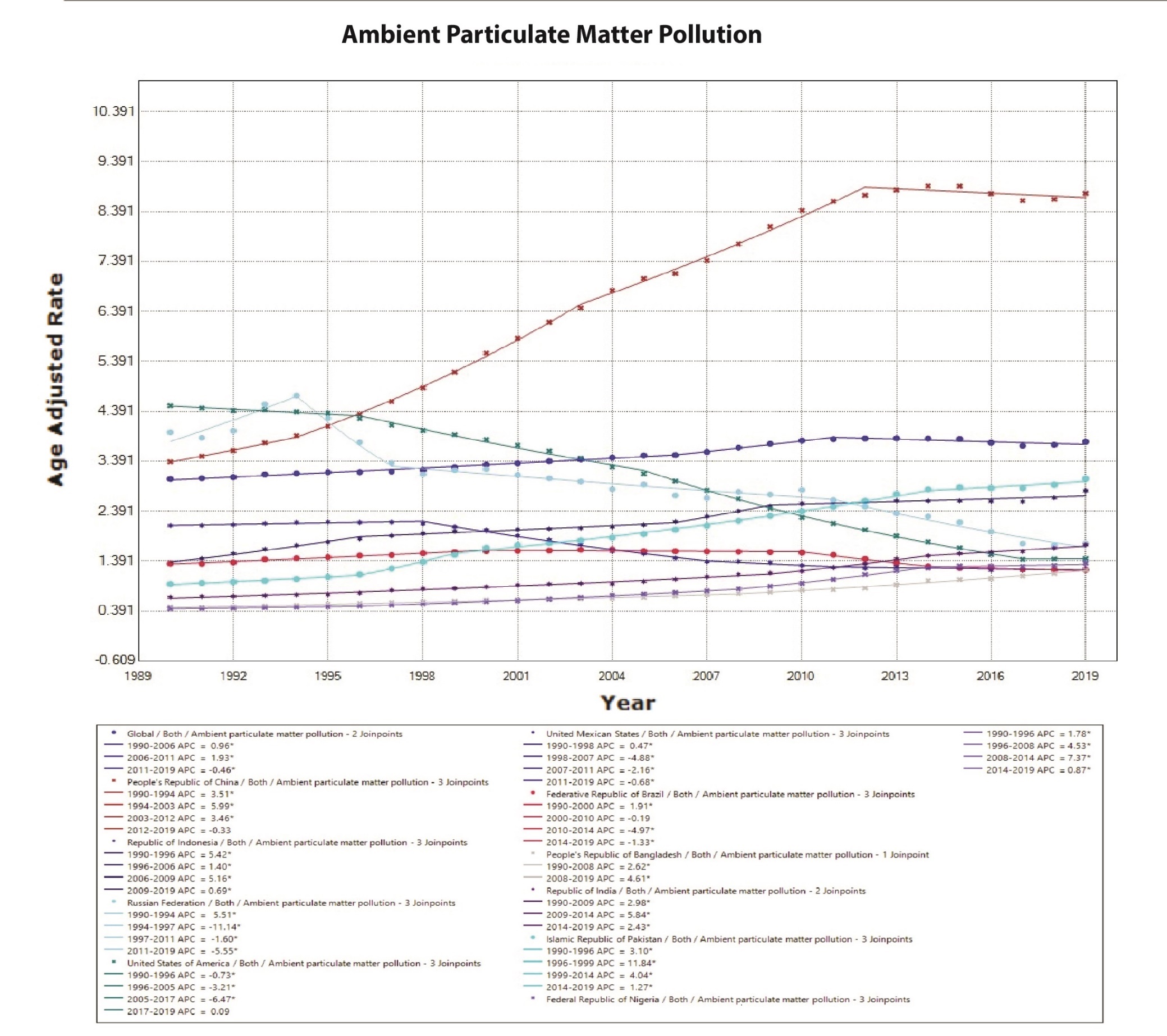


ASMR: Age Standardized Mortality Rate; APC: Annual Percentage Change

(D)


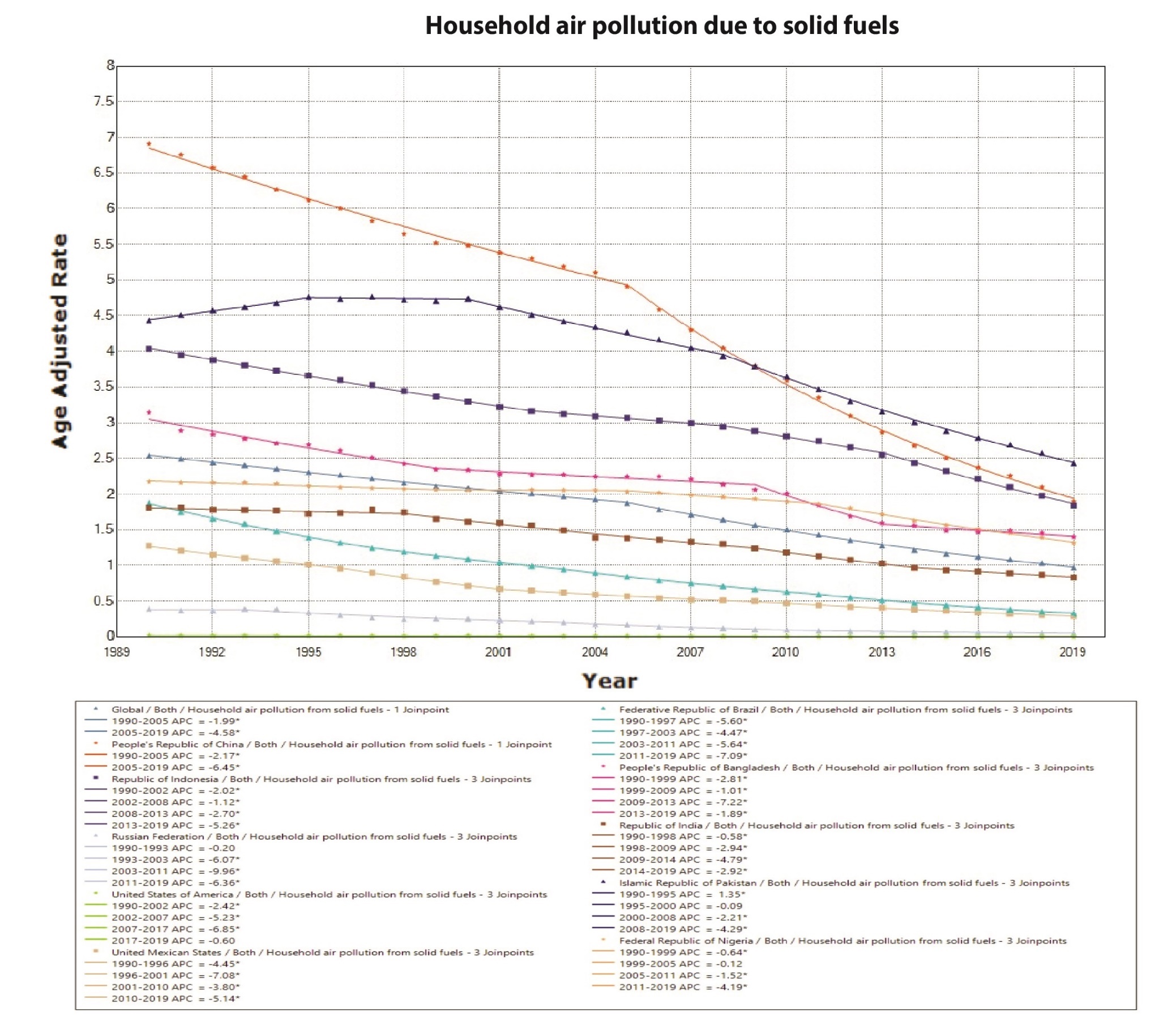


(E)

ASMR: Age Standardized Mortality Rate; APC: Annual Percentage Change


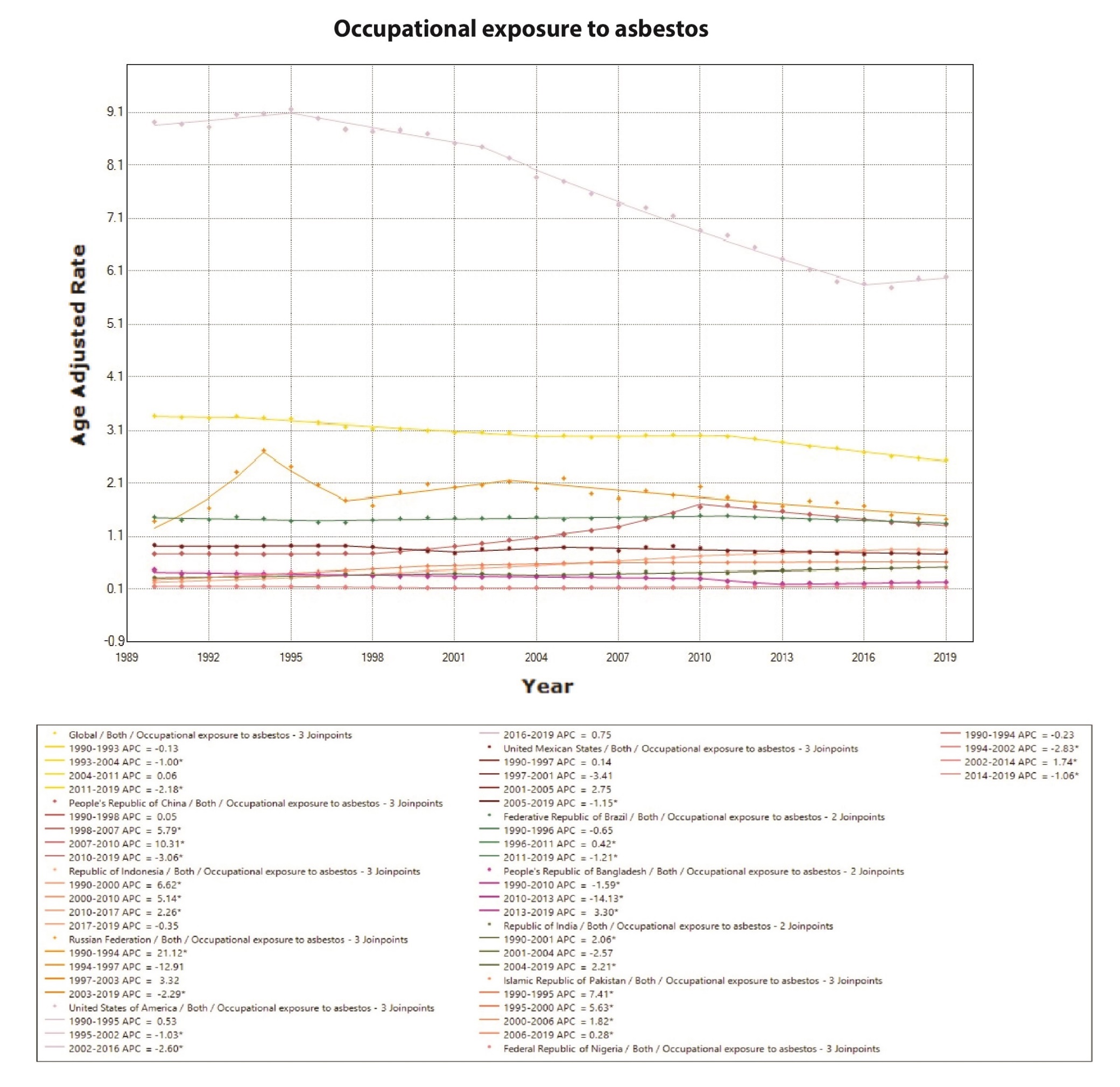


ASMR: Age Standardized Mortality Rate; APC: Annual Percentage Change

**Supplementary Table 1. Joinpoint analysis for TBL cancer mortality for the years 1990 to 2019 for both sexes combined based on risk factors: A. Air Pollution, B. Tobacco C. Occupational exposure to Asbestos D. Ambient Particulate Matter, E. Household air pollution**

| **Location** | **Trend 1** | | | **Trend 2** | | | **Trend 3** | | | **Trend 4** | | | **AAPC** |
| --- | --- | --- | --- | --- | --- | --- | --- | --- | --- | --- | --- | --- | --- |
|  | **Years** | **APC (95% CI)** | **p value** | **Years** | **APC (95% CI)** | **p value** | **Years** | **APC (95% CI)** | **p value** | **Years** | **APC (95% CI)** | **p value** |  |
| **Air Pollution** | | | | | | | | | | | | | |
| **Global** | 1990 -1998 | -0.4 (-0.5 - -0.2) | <0.001 | 1998 -2011 | -0.2 (-0.2 - -0.1) | <0.001 | 2011 -2017 | -1.6 (-1.9 - -1.3) | <0.001 | 2017 -2019 | -0.6 (-1.9 - 0.6) | 0.302 | -0.6 (-0.7 - -0.4) |
| **China** | 1990 -1996 | -0.1 (-0.4 - 0.3) | 0.684 | 1996 -2004 | 1.9 (1.6 - 2.2) | <0.001 | 2004 -2012 | 0 (-0.2 - 0.3) | 0.927 | 2012 -2019 | -1.6 (-1.9 - -1.3) | <0.001 | 0.1 (0 - 0.2) |
| **Indonesia** | 1990 -1997 | 0.2 (-0.1 - 0.4) | 0.217 | 1997 -2003 | -1 (-1.4 - -0.5) | <0.001 | 2003 -2011 | 0.5 (0.3 - 0.8) | <0.001 | 2011 -2019 | -2 (-2.2 - -1.8) | <0.001 | -0.6 (-0.7 - -0.4) |
| **Russia** | 1990 -1994 | 5.1 (1.5 - 8.8) | 0.008 | 1994 -1997 | -11 (-20.3 - -0.6) | 0.04 | 1997 -2011 | -1.9 (-2.5 - -1.4) | <0.001 | 2011 -2019 | -5.6 (-6.7 - -4.5) | <0.001 | -3 (-4.2 - -1.8) |
| **USA** | 1990 -1995 | -0.5 (-1 - 0) | 0.043 | 1995 -2004 | -2.8 (-3 - -2.6) | <0.001 | 2004 -2017 | -6.3 (-6.5 - -6.2) | <0.001 | 2017 -2019 | -0.1 (-2.3 - 2.2) | 0.921 | -3.8 (-4 - -3.7) |
| **Mexico** | 1990 -1998 | -1.4 (-1.9 - -0.9) | <0.001 | 1998 -2007 | -5.1 (-5.5 - -4.6) | <0.001 | 2007 -2019 | -2 (-2.2 - -1.7) | <0.001 |  |  |  | -2.8 (-3 - -2.6) |
| **Brazil** | 1990 -2004 | -1.6 (-1.7 - -1.5) | <0.001 | 2004 -2011 | -2.3 (-2.7 - -1.8) | <0.001 | 2011 -2014 | -6.2 (-8.7 - -3.6) | <0.001 | 2014 -2019 | -2.7 (-3.2 - -2.1) | <0.001 | -2.4 (-2.7 - -2.1) |
| **Bangladesh** | 1990 -1999 | -1.9 (-2.3 - -1.5) | <0.001 | 1999 -2009 | -0.2 (-0.6 - 0.2) | 0.236 | 2009 -2013 | -3.6 (-5.6 - -1.5) | 0.002 | 2013 -2019 | 0.8 (0.1 - 1.5) | 0.026 | -1 (-1.3 - -0.7) |
| **India** | 1990 -2001 | 0.2 (0 - 0.5) | 0.076 | 2001 -2004 | -2.5 (-6.3 - 1.5) | 0.204 | 2004 -2019 | 0.5 (0.3 - 0.6) | <0.001 |  |  |  | 0.1 (-0.3 - 0.5) |
| **Pakistan** | 1990 -2000 | 1.7 (1.6 - 1.8) | <0.001 | 2000 -2009 | -0.4 (-0.6 - -0.3) | <0.001 | 2009 -2019 | -1.1 (-1.2 - -1) | <0.001 |  |  |  | 0.1 (0 - 0.1) |
| **Nigeria** | 1990 -1997 | -0.2 (-0.4 - 0) | 0.121 | 1997 -2013 | 0.8 (0.7 - 0.8) | <0.001 | 2013 -2019 | -1.5 (-1.8 - -1.2) | <0.001 |  |  |  | 0.1 (0 - 0.1) |
| **Tobacco** | | | | | | | | | | | | | |
| **Global** | 1990 -1994 | 0.2 (-0.3 - 0.7) | 0.361 | 1994 -2010 | -0.5 (-0.6 - -0.4) | <0.001 | 2010 -2017 | -1.3 (-1.5 - -1.1) | <0.001 | 2017 -2019 | -0.3 (-1.7 - 1.2) | 0.712 | -0.6 (-0.7 - -0.4) |
| **China** | 1990 -1996 | 1.2 (0.8 - 1.5) | <0.001 | 1996 -2004 | 2.7 (2.4 - 3) | <0.001 | 2004 -2011 | 0.4 (0 - 0.7) | 0.03 | 2011 -2019 | -0.7 (-0.9 - -0.5) | <0.001 | 0.9 (0.7 - 1) |
| **Indonesia** | 1990 -1998 | 1.4 (1.3 - 1.4) | <0.001 | 1998 -2003 | 0.6 (0.4 - 0.7) | <0.001 | 2003 -2011 | 1.7 (1.6 - 1.8) | <0.001 | 2011 -2019 | 0.6 (0.6 - 0.7) | <0.001 | 1.1 (1.1 - 1.2) |
| **Russia** | 1990 -1994 | 7.8 (4.6 - 11) | <0.001 | 1994 -1997 | -9.8 (-17.8 - -1) | 0.031 | 1997 -2001 | 0.6 (-4 - 5.3) | 0.803 | 2001 -2019 | -1.7 (-2 - -1.4) | <0.001 | -1 (-2.1 - 0.2) |
| **USA** | 1990 -1995 | -0.2 (-0.6 - 0.2) | 0.356 | 1995 -2002 | -1.3 (-1.7 - -1) | <0.001 | 2002 -2017 | -2.2 (-2.3 - -2.2) | <0.001 | 2017 -2019 | 1.2 (-0.8 - 3.1) | 0.223 | -1.4 (-1.6 - -1.3) |
| **Mexico** | 1990 -1996 | -1.2 (-1.8 - -0.5) | 0.001 | 1996 -2016 | -3.5 (-3.6 - -3.4) | <0.001 | 2016 -2019 | 0.3 (-1.6 - 2.3) | 0.746 |  |  |  | -2.7 (-2.9 - -2.4) |
| **Brazil** | 1990 -2001 | -0.1 (-0.3 - 0) | 0.046 | 2001 -2016 | -1.7 (-1.8 - -1.6) | <0.001 | 2016 -2019 | -2.8 (-3.8 - -1.9) | <0.001 |  |  |  | -1.2 (-1.4 - -1.1) |
| **Bangladesh** | 1990 -2001 | -1.9 (-2.3 - -1.6) | <0.001 | 2001 -2008 | -0.1 (-0.9 - 0.7) | 0.847 | 2008 -2013 | -4 (-5.4 - -2.6) | <0.001 | 2013 -2019 | 1.1 (0.3 - 1.9) | 0.01 | -1.2 (-1.6 - -0.9) |
| **India** | 1990 -1998 | 0.7 (0.2 - 1.2) | 0.004 | 1998 -2005 | -1.7 (-2.4 - -1) | <0.001 | 2005 -2019 | 0 (-0.2 - 0.2) | 0.973 |  |  |  | -0.2 (-0.4 - 0) |
| **Pakistan** | 1990 -2000 | 1.8 (1.8 - 1.9) | <0.001 | 2000 -2008 | -0.3 (-0.4 - -0.2) | <0.001 | 2008 -2019 | -1.5 (-1.6 - -1.5) | <0.001 |  |  |  | 0 (-0.1 - 0) |
| **Nigeria** | 1990 -1998 | -0.3 (-0.4 - -0.2) | <0.001 | 1998 -2004 | 1.5 (1.3 - 1.7) | <0.001 | 2004 -2012 | 0.5 (0.4 - 0.6) | <0.001 | 2012 -2019 | -2 (-2.1 - -1.9) | <0.001 | -0.1 (-0.2 - -0.1) |
| **Occupational exposure to asbestos** | | | | | | | | | | | | | |
| **Global** | 1990 - 1993 | -0.1 (-1.3 - 1.1) | 0.823 | 1993 - 2004 | -1 (-1.2 - -0.8) | <0.001 | 2004 - 2011 | 0.1 (-0.3 - 0.5) | 0.759 | 2011 - 2019 | -2.2 (-2.4 - -1.9) | <0.001 | -1 (-1.2 - -0.8) |
| **China** | 1990 - 1998 | 0 (-0.3 - 0.4) | 0.796 | 1998 - 2007 | 5.8 (5.4 - 6.2) | <0.001 | 2007 - 2010 | 10.3 (6.5 - 14.2) | <0.001 | 2010 - 2019 | -3.1 (-3.4 - -2.8) | <0.001 | 1.8 (1.4 - 2.2) |
| **Indonesia** | 1990 - 2000 | 6.6 (6.5 - 6.7) | <0.001 | 2000 - 2010 | 5.1 (5 - 5.2) | <0.001 | 2010 - 2017 | 2.3 (2.1 - 2.4) | <0.001 | 2017 - 2019 | -0.4 (-1.4 - 0.7) | 0.498 | 4.6 (4.5 - 4.6) |
| **Russia** | 1990 - 1994 | 21.1 (13.8 - 28.9) | <0.001 | 1994 - 1997 | -12.9 (-28.5 - 6.1) | 0.16 | 1997 - 2003 | 3.3 (-1.1 - 8) | 0.139 | 2003 - 2019 | -2.3 (-3 - -1.5) | <0.001 | 0.6 (-1.7 - 2.9) |
| **USA** | 1990 - 1995 | 0.5 (-0.2 - 1.2) | 0.13 | 1995 - 2002 | -1 (-1.6 - -0.5) | 0.001 | 2002 - 2016 | -2.6 (-2.8 - -2.4) | <0.001 | 2016 - 2019 | 0.8 (-0.8 - 2.3) | 0.328 | -1.3 (-1.6 - -1.1) |
| **Mexico** | 1990 - 1997 | 0.1 (-1 - 1.3) | 0.801 | 1997 - 2001 | -3.4 (-7.6 - 1) | 0.119 | 2001 - 2005 | 2.8 (-1.7 - 7.4) | 0.216 | 2005 - 2019 | -1.2 (-1.6 - -0.7) | <0.001 | -0.6 (-1.5 - 0.2) |
| **Brazil** | 1990 - 1996 | -0.7 (-1.5 - 0.2) | 0.132 | 1996 - 2011 | 0.4 (0.2 - 0.7) | 0.001 | 2011 - 2019 | -1.2 (-1.8 - -0.7) | <0.001 |  |  |  | -0.3 (-0.5 - 0) |
| **Bangladesh** | 1990 - 2010 | -1.6 (-1.9 - -1.2) | <0.001 | 2010 - 2013 | -14.1 (-24.5 - -2.3) | 0.022 | 2013 - 2019 | 3.3 (1.1 - 5.6) | 0.005 |  |  |  | -2 (-3.3 - -0.7) |
| **India** | 1990 - 2001 | 2.1 (1.4 - 2.7) | <0.001 | 2001 - 2004 | -2.6 (-11.8 - 7.6) | 0.592 | 2004 - 2019 | 2.2 (1.8 - 2.6) | <0.001 |  |  |  | 1.6 (0.6 - 2.7) |
| **Pakistan** | 1990 - 1995 | 7.4 (6.9 - 8) | <0.001 | 1995 - 2000 | 5.6 (4.9 - 6.4) | <0.001 | 2000 - 2006 | 1.8 (1.3 - 2.3) | <0.001 | 2006 - 2019 | 0.3 (0.2 - 0.4) | <0.001 | 2.7 (2.5 - 2.9) |
| **Nigeria** | 1990 - 1994 | -0.2 (-1.3 - 0.8) | 0.648 | 1994 - 2002 | -2.8 (-3.3 - -2.4) | <0.001 | 2002 - 2014 | 1.7 (1.5 - 2) | <0.001 | 2014 - 2019 | -1.1 (-1.8 - -0.3) | 0.007 | -0.3 (-0.5 - -0.1) |
| **Ambient Particulate Matter Pollution** | | | | | | | | | | | | | |
| **Global** | 1990 - 2006 | 1 (0.9 - 1.1) | <0.001 | 2006 - 2011 | 1.9 (1.2 - 2.7) | <0.001 | 2011 - 2019 | -0.5 (-0.7 - -0.2) | 0.001 |  |  |  | 0.7 (0.6 - 0.9) |
| **China** | 1990 - 1994 | 3.5 (2.7 - 4.3) | <0.001 | 1994 - 2003 | 6 (5.7 - 6.3) | <0.001 | 2003 - 2012 | 3.5 (3.2 - 3.7) | <0.001 | 2012 - 2019 | -0.3 (-0.7 - 0) | 0.051 | 3.3 (3.1 - 3.5) |
| **Indonesia** | 1990 - 1996 | 5.4 (4.6 - 6.2) | <0.001 | 1996 - 2006 | 1.4 (1 - 1.8) | <0.001 | 2006 - 2009 | 5.2 (0.6 - 9.9) | 0.027 | 2009 - 2019 | 0.7 (0.3 - 1) | <0.001 | 2.4 (1.9 - 2.9) |
| **Russia** | 1990 - 1994 | 5.5 (1.8 - 9.4) | 0.005 | 1994 - 1997 | -11.1 (-20.6 - -0.5) | 0.042 | 1997 - 2011 | -1.6 (-2.2 - -1) | <0.001 | 2011 - 2019 | -5.5 (-6.7 - -4.4) | <0.001 | -2.8 (-4 - -1.6) |
| **USA** | 1990 - 1996 | -0.7 (-1.2 - -0.3) | 0.003 | 1996 - 2005 | -3.2 (-3.5 - -2.9) | <0.001 | 2005 - 2017 | -6.5 (-6.6 - -6.3) | <0.001 | 2017 - 2019 | 0.1 (-2.5 - 2.8) | 0.943 | -3.9 (-4.1 - -3.6) |
| **Mexico** | 1990 - 1998 | 0.5 (0 - 0.9) | 0.046 | 1998 - 2007 | -4.9 (-5.3 - -4.4) | <0.001 | 2007 - 2011 | -2.2 (-4.2 - -0.1) | 0.042 | 2011 - 2019 | -0.7 (-1.1 - -0.2) | 0.005 | -1.9 (-2.2 - -1.6) |
| **Brazil** | 1990 - 2000 | 1.9 (1.7 - 2.1) | <0.001 | 2000 - 2010 | -0.2 (-0.4 - 0) | 0.102 | 2010 - 2014 | -5 (-6.2 - -3.7) | <0.001 | 2014 - 2019 | -1.3 (-1.9 - -0.8) | <0.001 | -0.3 (-0.6 - -0.1) |
| **Bangladesh** | 1990 - 2008 | 2.6 (2.4 - 2.8) | <0.001 | 2008 - 2019 | 4.6 (4.2 - 5) | <0.001 |  |  |  |  |  |  | 3.4 (3.2 - 3.6) |
| **India** | 1990 - 2009 | 3 (2.8 - 3.2) | <0.001 | 2009 - 2014 | 5.8 (3.8 - 7.9) | <0.001 | 2014 - 2019 | 2.4 (1 - 3.8) | 0.001 |  |  |  | 3.4 (3 - 3.8) |
| **Pakistan** | 1990 - 1996 | 3.1 (2.4 - 3.8) | <0.001 | 1996 - 1999 | 11.8 (7.5 - 16.4) | <0.001 | 1999 - 2014 | 4 (3.8 - 4.2) | <0.001 | 2014 - 2019 | 1.3 (0.4 - 2.2) | 0.008 | 4.1 (3.7 - 4.6) |
| **Nigeria** | 1990 - 1996 | 1.8 (1.3 - 2.2) | <0.001 | 1996 - 2008 | 4.5 (4.3 - 4.7) | <0.001 | 2008 - 2014 | 7.4 (6.7 - 8) | <0.001 | 2014 - 2019 | 0.9 (0.3 - 1.5) | 0.006 | 3.9 (3.7 - 4.1) |
| **Household air pollution from solid fuels** | | | | | | | | | | | | | |
| **Global** | 1990 - 2005 | -2 (-2.1 - -1.9) | <0.001 | 2005 - 2019 | -4.6 (-4.7 - -4.5) | <0.001 |  |  |  |  |  |  | -3.3 (-3.3 - -3.2) |
| **China** | 1990 - 2005 | -2.2 (-2.3 - -2) | <0.001 | 2005 - 2019 | -6.4 (-6.6 - -6.3) | <0.001 |  |  |  |  |  |  | -4.3 (-4.4 - -4.2) |
| **Indonesia** | 1990 - 2002 | -2 (-2.1 - -1.9) | <0.001 | 2002 - 2008 | -1.1 (-1.4 - -0.8) | <0.001 | 2008 - 2013 | -2.7 (-3.2 - -2.2) | <0.001 | 2013 - 2019 | -5.3 (-5.5 - -5) | <0.001 | -2.6 (-2.7 - -2.5) |
| **Russia** | 1990 - 1993 | -0.2 (-6.6 - 6.6) | 0.95 | 1993 - 2003 | -6.1 (-7.2 - -4.9) | <0.001 | 2003 - 2011 | -10 (-11.5 - -8.4) | <0.001 | 2011 - 2019 | -6.4 (-7.7 - -5) | <0.001 | -6.7 (-7.5 - -5.8) |
| **USA** | 1990 - 2002 | -2.4 (-2.5 - -2.3) | <0.001 | 2002 - 2007 | -5.2 (-5.7 - -4.7) | <0.001 | 2007 - 2017 | -6.8 (-7 - -6.7) | <0.001 | 2017 - 2019 | -0.6 (-2.2 - 1) | 0.439 | -4.3 (-4.5 - -4.2) |
| **Mexico** | 1990 - 1996 | -4.5 (-5 - -3.9) | <0.001 | 1996 - 2001 | -7.1 (-8.1 - -6) | <0.001 | 2001 - 2010 | -3.8 (-4.2 - -3.4) | <0.001 | 2010 - 2019 | -5.1 (-5.4 - -4.8) | <0.001 | -4.9 (-5.2 - -4.7) |
| **Brazil** | 1990 - 1997 | -5.6 (-5.8 - -5.4) | <0.001 | 1997 - 2003 | -4.5 (-4.8 - -4.2) | <0.001 | 2003 - 2011 | -5.6 (-5.8 - -5.5) | <0.001 | 2011 - 2019 | -7.1 (-7.2 - -7) | <0.001 | -5.8 (-5.9 - -5.7) |
| **Bangladesh** | 1990 - 1999 | -2.8 (-3.2 - -2.4) | <0.001 | 1999 - 2009 | -1 (-1.4 - -0.6) | <0.001 | 2009 - 2013 | -7.2 (-9.3 - -5.1) | <0.001 | 2013 - 2019 | -1.9 (-2.6 - -1.1) | <0.001 | -2.6 (-3 - -2.3) |
| **India** | 1990 - 1998 | -0.6 (-1 - -0.1) | 0.017 | 1998 - 2009 | -2.9 (-3.3 - -2.6) | <0.001 | 2009 - 2014 | -4.8 (-6.1 - -3.5) | <0.001 | 2014 - 2019 | -2.9 (-3.8 - -2) | <0.001 | -2.6 (-2.9 - -2.3) |
| **Pakistan** | 1990 - 1995 | 1.3 (0.9 - 1.8) | <0.001 | 1995 - 2000 | -0.1 (-0.7 - 0.5) | 0.748 | 2000 - 2008 | -2.2 (-2.4 - -2) | <0.001 | 2008 - 2019 | -4.3 (-4.4 - -4.2) | <0.001 | -2 (-2.2 - -1.9) |
| **Nigeria** | 1990 - 1999 | -0.6 (-0.8 - -0.5) | <0.001 | 1999 - 2005 | -0.1 (-0.5 - 0.2) | 0.476 | 2005 - 2011 | -1.5 (-1.9 - -1.2) | <0.001 | 2011 - 2019 | -4.2 (-4.4 - -4) | <0.001 | -1.7 (-1.8 - -1.6) |

APC: Annual Percentage Change; AAPC: Average Annual Percentage Change; ASMR: Age Standardized Mortality Rate; US: United States.

**Supplementary table 2. Joinpoint analysis for TBL cancer mortality for years 1990 to 2019 for Males based on risk factors: A. Air Pollution, B. Tobacco C. Occupational exposure to Asbestos D. Ambient Particulate Matter, E. Household air pollution**

| **Location** | **Trend 1** | | | **Trend 2** | | | **Trend 3** | | | **Trend 4** | | | **AAPC** |
| --- | --- | --- | --- | --- | --- | --- | --- | --- | --- | --- | --- | --- | --- |
|  | **Years** | **APC (95% CI)** | **p value** | **Years** | **APC (95% CI)** | **p value** | **Years** | **APC (95% CI)** | **p value** | **Years** | **APC (95% CI)** | **p value** |  |
| **Air Pollution** | | | | | | | | | | | | | |
| **Global** | 1990 - 1998 | -0.8 (-0.9 - -0.6) | <0.001 | 1998 - 2012 | -0.4 (-0.5 - -0.3) | <0.001 | 2012 - 2019 | -1.9 (-2.1 - -1.7) | <0.001 |  |  |  | -0.9 (-0.9 - -0.8) |
| **China** | 1990 - 1995 | -0.4 (-1 - 0.2) | 0.171 | 1995 - 2004 | 1.8 (1.5 - 2.1) | <0.001 | 2004 - 2013 | 0.5 (0.2 - 0.8) | 0.001 | 2013 - 2019 | -2.3 (-2.7 - -1.8) | <0.001 | 0.2 (0 - 0.3) |
| **Indonesia** | 1990 - 2004 | -0.7 (-0.7 - -0.6) | <0.001 | 2004 - 2011 | 0.8 (0.5 - 1) | <0.001 | 2011 - 2017 | -2.6 (-2.9 - -2.2) | <0.001 | 2017 - 2019 | -0.7 (-2.4 - 0.9) | 0.359 | -0.7 (-0.9 - -0.6) |
| **Russia** | 1990 - 1994 | 4.8 (0.9 - 8.7) | 0.017 | 1994 - 1997 | -11.3 (-21.1 - -0.2) | 0.047 | 1997 - 2011 | -2.2 (-2.8 - -1.6) | <0.001 | 2011 - 2019 | -6.3 (-7.5 - -5.1) | <0.001 | -3.4 (-4.6 - -2.1) |
| **USA** | 1990 - 1995 | -1.5 (-2 - -1.1) | <0.001 | 1995 - 2004 | -3.9 (-4.1 - -3.7) | <0.001 | 2004 - 2017 | -6.8 (-6.9 - -6.7) | <0.001 | 2017 - 2019 | 0.7 (-1.4 - 2.8) | 0.515 | -4.5 (-4.7 - -4.3) |
| **Mexico** | 1990 - 1998 | -1.3 (-1.7 - -0.9) | <0.001 | 1998 - 2007 | -5.1 (-5.5 - -4.7) | <0.001 | 2007 - 2017 | -2.3 (-2.7 - -1.9) | <0.001 | 2017 - 2019 | 0.4 (-3.5 - 4.5) | 0.825 | -2.7 (-3.1 - -2.4) |
| **Brazil** | 1990 - 2004 | -1.9 (-2.1 - -1.8) | <0.001 | 2004 - 2011 | -3.2 (-3.7 - -2.8) | <0.001 | 2011 - 2014 | -7.2 (-9.8 - -4.6) | <0.001 | 2014 - 2019 | -3.1 (-3.7 - -2.5) | <0.001 | -3 (-3.3 - -2.7) |
| **Bangladesh** | 1990 - 2000 | -2.3 (-2.6 - -1.9) | <0.001 | 2000 - 2009 | 0 (-0.5 - 0.5) | 0.964 | 2009 - 2013 | -4.2 (-6.2 - -2.1) | 0.001 | 2013 - 2019 | 0.7 (0 - 1.5) | 0.049 | -1.2 (-1.6 - -0.9) |
| **India** | 1990 - 1998 | 0.4 (0 - 0.8) | 0.051 | 1998 - 2004 | -1.1 (-1.9 - -0.3) | 0.007 | 2004 - 2019 | 0 (-0.2 - 0.1) | 0.544 |  |  |  | -0.2 (-0.3 - 0) |
| **Pakistan** | 1990 - 2000 | 1.9 (1.8 - 2) | <0.001 | 2000 - 2010 | -0.5 (-0.6 - -0.4) | <0.001 | 2010 - 2019 | -1.4 (-1.5 - -1.2) | <0.001 |  |  |  | 0.1 (0 - 0.1) |
| **Nigeria** | 1990 - 1997 | -0.9 (-1 - -0.7) | <0.001 | 1997 - 2008 | 0.3 (0.2 - 0.4) | <0.001 | 2008 - 2013 | 0.6 (0.2 - 1) | 0.007 | 2013 - 2019 | -1.7 (-1.9 - -1.5) | <0.001 | -0.4 (-0.5 - -0.3) |
| **Tobacco** | | | | | | | | | | | | | |
| **Global** | 1990 - 1994 | 0 (-0.5 - 0.5) | 0.919 | 1994 - 1997 | -1.2 (-2.7 - 0.3) | 0.104 | 1997 - 2010 | -0.7 (-0.8 - -0.6) | <0.001 | 2010 - 2019 | -1.3 (-1.4 - -1.1) | <0.001 | -0.8 (-1 - -0.7) |
| **China** | 1990 - 1995 | 0.6 (0.1 - 1.1) | 0.018 | 1995 - 2004 | 2.4 (2.1 - 2.6) | <0.001 | 2004 - 2011 | 0.9 (0.5 - 1.3) | <0.001 | 2011 - 2019 | -0.7 (-0.9 - -0.4) | <0.001 | 0.9 (0.7 - 1) |
| **Indonesia** | 1990 - 1997 | 1.3 (1.2 - 1.4) | <0.001 | 1997 - 2003 | 0.7 (0.5 - 0.8) | <0.001 | 2003 - 2011 | 1.7 (1.7 - 1.8) | <0.001 | 2011 - 2019 | 0.5 (0.4 - 0.6) | <0.001 | 1.1 (1 - 1.1) |
| **Russia** | 1990 - 1994 | 6.8 (3.5 - 10.1) | <0.001 | 1994 - 1997 | -9.6 (-17.9 - -0.5) | 0.041 | 1997 - 2002 | 0 (-3 - 3.1) | 0.985 | 2002 - 2019 | -2.1 (-2.4 - -1.7) | <0.001 | -1.4 (-2.5 - -0.2) |
| **USA** | 1990 - 1995 | -1.2 (-1.7 - -0.8) | <0.001 | 1995 - 2001 | -2.4 (-2.8 - -2) | <0.001 | 2001 - 2016 | -2.8 (-2.9 - -2.7) | <0.001 | 2016 - 2019 | 0.5 (-0.5 - 1.5) | 0.345 | -2.1 (-2.2 - -1.9) |
| **Mexico** | 1990 - 1996 | -0.9 (-1.7 - -0.2) | 0.014 | 1996 - 2016 | -3.4 (-3.5 - -3.3) | <0.001 | 2016 - 2019 | 1.1 (-1.1 - 3.3) | 0.305 |  |  |  | -2.4 (-2.7 - -2.2) |
| **Brazil** | 1990 - 2002 | -0.4 (-0.6 - -0.3) | <0.001 | 2002 - 2019 | -2.5 (-2.6 - -2.4) | <0.001 |  |  |  |  |  |  | -1.6 (-1.7 - -1.5) |
| **Bangladesh** | 1990 - 2001 | -2.1 (-2.4 - -1.8) | <0.001 | 2001 - 2009 | -0.1 (-0.7 - 0.5) | 0.809 | 2009 - 2013 | -4.4 (-6.5 - -2.3) | <0.001 | 2013 - 2019 | 1.4 (0.7 - 2.2) | 0.001 | -1.1 (-1.5 - -0.8) |
| **India** | 1990 - 1998 | 0.8 (0.5 - 1.1) | <0.001 | 1998 - 2004 | -1.6 (-2.3 - -0.9) | <0.001 | 2004 - 2019 | -0.1 (-0.3 - 0) | 0.034 |  |  |  | -0.2 (-0.4 - 0) |
| **Pakistan** | 1990 - 2000 | 2.2 (2.1 - 2.2) | <0.001 | 2000 - 2009 | -0.3 (-0.4 - -0.2) | <0.001 | 2009 - 2019 | -1.5 (-1.6 - -1.5) | <0.001 |  |  |  | 0.1 (0.1 - 0.2) |
| **Nigeria** | 1990 - 1997 | -1.5 (-1.7 - -1.3) | <0.001 | 1997 - 2013 | 0.7 (0.7 - 0.8) | <0.001 | 2013 - 2019 | -1.5 (-1.7 - -1.2) | <0.001 |  |  |  | -0.3 (-0.3 - -0.2) |
| **Occupational exposure to asbestos** | | | | | | | | | | | | | |
| **Global** | 1990 -1993 | -0.4 (-1.6 - 0.7) | 0.445 | 1993 -2004 | -1.3 (-1.4 - -1.1) | <0.001 | 2004 -2011 | -0.1 (-0.5 - 0.3) | 0.488 | 2011 -2019 | -2.5 (-2.7 - -2.2) | <0.001 | -1.2 (-1.4 - -1.1) |
| **China** | 1990 -1998 | 0.5 (0.1 - 0.9) | 0.01 | 1998 -2005 | 6.4 (5.7 - 7) | <0.001 | 2005 -2011 | 9.2 (8.3 - 10.1) | <0.001 | 2011 -2019 | -4.3 (-4.6 - -3.9) | <0.001 | 2.3 (2 - 2.5) |
| **Indonesia** | 1990 -2000 | 7.2 (7 - 7.3) | <0.001 | 2000 -2010 | 5.8 (5.6 - 6) | <0.001 | 2010 -2017 | 2.7 (2.4 - 3.1) | <0.001 | 2017 -2019 | -0.4 (-2.2 - 1.5) | 0.661 | 5.1 (4.9 - 5.2) |
| **Russia** | 1990 -1994 | 20.5 (12.7 - 28.9) | <0.001 | 1994 -1997 | -12.7 (-29.5 - 8) | 0.196 | 1997 -2003 | 2.8 (-2 - 7.8) | 0.241 | 2003 -2019 | -2.5 (-3.3 - -1.7) | <0.001 | 0.3 (-2.1 - 2.8) |
| **USA** | 1990 -1995 | 0 (-0.7 - 0.8) | 0.937 | 1995 -2002 | -1.6 (-2.1 - -1.1) | <0.001 | 2002 -2016 | -3.1 (-3.2 - -2.9) | <0.001 | 2016 -2019 | 0.8 (-0.8 - 2.5) | 0.293 | -1.8 (-2 - -1.6) |
| **Mexico** | 1990 -2009 | 0 (-0.4 - 0.3) | 0.867 | 2009 -2019 | -1.1 (-1.9 - -0.2) | 0.021 |  |  |  |  |  |  | -0.4 (-0.7 - 0) |
| **Brazil** | 1990 -1997 | -1 (-1.7 - -0.2) | 0.015 | 1997 -2000 | 2.3 (-3.4 - 8.4) | 0.411 | 2000 -2011 | 0.2 (-0.3 - 0.6) | 0.446 | 2011 -2019 | -1.7 (-2.3 - -1.1) | <0.001 | -0.4 (-1 - 0.2) |
| **Bangladesh** | 1990 -2001 | -2.6 (-3.4 - -1.8) | <0.001 | 2001 -2009 | -0.2 (-1.8 - 1.4) | 0.795 | 2009 -2013 | -12.9 (-17.9 - -7.6) | <0.001 | 2013 -2019 | 3 (0.9 - 5.1) | 0.006 | -2.3 (-3.3 - -1.3) |
| **India** | 1990 -2001 | 2.2 (1.5 - 2.9) | <0.001 | 2001 -2004 | -1.9 (-11.6 - 8.7) | 0.698 | 2004 -2019 | 2.6 (2.2 - 3.1) | <0.001 |  |  |  | 2 (0.9 - 3.1) |
| **Pakistan** | 1990 -1995 | 8.1 (7.4 - 8.8) | <0.001 | 1995 -2000 | 6.1 (5.1 - 7.1) | <0.001 | 2000 -2006 | 2.2 (1.5 - 2.9) | <0.001 | 2006 -2019 | 0.4 (0.3 - 0.6) | <0.001 | 3 (2.8 - 3.3) |
| **Nigeria** | 1990 -1994 | -0.7 (-2.4 - 1.1) | 0.431 | 1994 -2002 | -5 (-5.7 - -4.3) | <0.001 | 2002 -2014 | 1.8 (1.4 - 2.2) | <0.001 | 2014 -2019 | -1.2 (-2.4 - 0) | 0.051 | -1 (-1.3 - -0.6) |
| **Ambient Particulate Matter Pollution** | | | | | | | | | | | | | |
| **Global** | 1990 -1998 | 0.2 (0 - 0.5) | 0.029 | 1998 -2006 | 0.8 (0.5 - 1.1) | <0.001 | 2006 -2012 | 1.5 (1 - 1.9) | <0.001 | 2012 -2019 | -1.1 (-1.3 - -0.8) | <0.001 | 0.3 (0.2 - 0.5) |
| **China** | 1990 -1994 | 3.3 (2.3 - 4.2) | <0.001 | 1994 -2003 | 5.8 (5.4 - 6.1) | <0.001 | 2003 -2013 | 3.5 (3.3 - 3.8) | <0.001 | 2013 -2019 | -1.3 (-1.8 - -0.8) | <0.001 | 3.2 (3 - 3.4) |
| **Indonesia** | 1990 -1996 | 4.7 (4 - 5.5) | <0.001 | 1996 -2005 | 1.2 (0.8 - 1.7) | <0.001 | 2005 -2010 | 3.8 (2.5 - 5.2) | <0.001 | 2010 -2019 | 0.2 (-0.1 - 0.6) | 0.218 | 2.1 (1.8 - 2.4) |
| **Russia** | 1990 -1994 | 5.1 (1.2 - 9.1) | 0.012 | 1994 -1997 | -11.4 (-21.3 - -0.2) | 0.047 | 1997 -2011 | -1.8 (-2.4 - -1.2) | <0.001 | 2011 -2019 | -6.3 (-7.5 - -5.1) | <0.001 | -3.2 (-4.5 - -1.9) |
| **USA** | 1990 -1995 | -1.5 (-1.9 - -1) | <0.001 | 1995 -2004 | -3.9 (-4.1 - -3.7) | <0.001 | 2004 -2017 | -6.8 (-6.9 - -6.7) | <0.001 | 2017 -2019 | 0.4 (-1.7 - 2.5) | 0.72 | -4.5 (-4.7 - -4.3) |
| **Mexico** | 1990 -1998 | 0.5 (-0.1 - 1) | 0.081 | 1998 -2007 | -5.1 (-5.6 - -4.6) | <0.001 | 2007 -2017 | -1.7 (-2.1 - -1.2) | <0.001 | 2017 -2019 | 2.1 (-2.8 - 7.2) | 0.393 | -1.9 (-2.3 - -1.5) |
| **Brazil** | 1990 -2001 | 1.4 (1.1 - 1.6) | <0.001 | 2001 -2010 | -1.2 (-1.6 - -0.8) | <0.001 | 2010 -2014 | -5.9 (-7.6 - -4.3) | <0.001 | 2014 -2019 | -2 (-2.7 - -1.2) | <0.001 | -1 (-1.3 - -0.7) |
| **Bangladesh** | 1990 -1992 | -1.6 (-6.3 - 3.4) | 0.505 | 1992 -1996 | 3.9 (1.4 - 6.5) | 0.004 | 1996 -2005 | 1.8 (1.3 - 2.4) | <0.001 | 2005 -2019 | 4.2 (4 - 4.4) | <0.001 | 3 (2.5 - 3.5) |
| **India** | 1990 -2006 | 2.7 (2.5 - 3) | <0.001 | 2006 -2014 | 4.3 (3.5 - 5.2) | <0.001 | 2014 -2019 | 1.8 (0.4 - 3.2) | 0.014 |  |  |  | 3 (2.7 - 3.4) |
| **Pakistan** | 1990 -1995 | 2.5 (1.8 - 3.3) | <0.001 | 1995 -2000 | 9.3 (8.2 - 10.4) | <0.001 | 2000 -2014 | 3.9 (3.7 - 4) | <0.001 | 2014 -2019 | 1 (0.3 - 1.7) | 0.01 | 4 (3.8 - 4.3) |
| **Nigeria** | 1990 -1996 | 0.3 (-0.3 - 1) | 0.323 | 1996 -2008 | 4 (3.8 - 4.3) | <0.001 | 2008 -2014 | 6.9 (6 - 7.8) | <0.001 | 2014 -2019 | 0.5 (-0.3 - 1.4) | 0.227 | 3.2 (3 - 3.5) |
| **Household air pollution from solid fuels** | | | | | | | | | | | | | |
| **Global** | 1990 -2005 | -2.3 (-2.4 - -2.2) | <0.001 | 2005 -2017 | -4.9 (-5 - -4.7) | <0.001 | 2017 -2019 | -7.2 (-9.1 - -5.2) | <0.001 |  |  |  | -3.7 (-3.8 - -3.6) |
| **China** | 1990 -1998 | -2.6 (-2.9 - -2.4) | <0.001 | 1998 -2005 | -2.1 (-2.5 - -1.7) | <0.001 | 2005 -2017 | -6.4 (-6.5 - -6.2) | <0.001 | 2017 -2019 | -9.6 (-11.8 - -7.3) | <0.001 | -4.6 (-4.8 - -4.4) |
| **Indonesia** | 1990 -2000 | -2.6 (-2.8 - -2.5) | <0.001 | 2000 -2010 | -1.4 (-1.5 - -1.3) | <0.001 | 2010 -2016 | -4.3 (-4.6 - -4.1) | <0.001 | 2016 -2019 | -7.2 (-7.8 - -6.6) | <0.001 | -3.1 (-3.2 - -3) |
| **Russia** | 1990 -2003 | -5.5 (-6.4 - -4.6) | <0.001 | 2003 -2011 | -10.6 (-12.7 - -8.5) | <0.001 | 2011 -2019 | -7.1 (-8.9 - -5.3) | <0.001 |  |  |  | -7.4 (-8.2 - -6.6) |
| **USA** | N/A | N/A | N/A | N/A | N/A | N/A | N/A | N/A | N/A | N/A | N/A | N/A | N/A |
| **Mexico** | 1990 -1997 | -4.7 (-5.3 - -4) | <0.001 | 1997 -2001 | -7.9 (-10.2 - -5.5) | <0.001 | 2001 -2010 | -3.8 (-4.3 - -3.2) | <0.001 | 2010 -2019 | -5.1 (-5.6 - -4.7) | <0.001 | -5 (-5.4 - -4.6) |
| **Brazil** | 1990 -1997 | -6.2 (-6.7 - -5.7) | <0.001 | 1997 -2003 | -5 (-5.8 - -4.2) | <0.001 | 2003 -2010 | -6.7 (-7.3 - -6.1) | <0.001 | 2010 -2019 | -7.8 (-8.1 - -7.5) | <0.001 | -6.6 (-6.8 - -6.3) |
| **Bangladesh** | 1990 -2000 | -3.2 (-3.6 - -2.9) | <0.001 | 2000 -2009 | -0.8 (-1.3 - -0.3) | 0.004 | 2009 -2013 | -8.1 (-10.4 - -5.9) | <0.001 | 2013 -2019 | -2.1 (-2.9 - -1.3) | <0.001 | -3 (-3.4 - -2.6) |
| **India** | 1990 -1998 | -0.8 (-1.3 - -0.2) | 0.006 | 1998 -2010 | -3.2 (-3.5 - -2.8) | <0.001 | 2010 -2015 | -6.1 (-7.5 - -4.6) | <0.001 | 2015 -2019 | -3.7 (-5.1 - -2.2) | <0.001 | -3.1 (-3.4 - -2.7) |
| **Pakistan** | 1990 -1995 | 1.6 (1.1 - 2) | <0.001 | 1995 -2000 | -0.1 (-0.7 - 0.6) | 0.855 | 2000 -2008 | -2.3 (-2.5 - -2) | <0.001 | 2008 -2019 | -4.6 (-4.8 - -4.5) | <0.001 | -2.2 (-2.3 - -2) |
| **Nigeria** | 1990 -1998 | -1.3 (-1.6 - -1.1) | <0.001 | 1998 -2004 | -0.6 (-1.1 - -0.1) | 0.018 | 2004 -2011 | -1.8 (-2.1 - -1.4) | <0.001 | 2011 -2019 | -4.3 (-4.5 - -4.1) | <0.001 | -2.1 (-2.3 - -2) |

APC: Annual Percentage Change; AAPC: Average Annual Percentage Change; ASMR: Age Standardized Mortality Rate; N/A: Not available for the US due to a small sample size.; US: United States.

**Supplementary table 3. Joinpoint analysis for TBL cancer mortality for years 1990 to 2019 for Females based on risk factors: A. Air Pollution, B. Tobacco C. Occupational exposure to Asbestos D. Ambient Particulate Matter, E. Household air pollution**

| **Location** | **Trend 1** | | | **Trend 2** | | | **Trend 3** | | | **Trend 4** | | | **AAPC** |
| --- | --- | --- | --- | --- | --- | --- | --- | --- | --- | --- | --- | --- | --- |
|  | **Years** | **APC (95% CI)** | **p value** | **Years** | **APC (95% CI)** | **p value** | **Years** | **APC (95% CI)** | **p value** | **Years** | **APC (95% CI)** | **p value** |  |
| **Air Pollution** | | | | | | | | | | | | | |
| **Global** | 1990 - 2004 | 0.5 (0.5 - 0.6) | <0.001 | 2004 - 2010 | -0.2 (-0.5 - 0.1) | 0.281 | 2010 - 2017 | -1 (-1.2 - -0.7) | <0.001 | 2017 - 2019 | 1.3 (-0.1 - 2.7) | 0.073 | 0.1 (-0.1 - 0.2) |
| **China** | 1990 - 1998 | 0.2 (-0.1 - 0.4) | 0.234 | 1998 - 2004 | 2.3 (1.7 - 2.9) | <0.001 | 2004 - 2017 | -1.1 (-1.2 - -0.9) | <0.001 | 2017 - 2019 | 0.8 (-1.8 - 3.5) | 0.517 | 0.1 (-0.1 - 0.3) |
| **Indonesia** | 1990 - 1997 | 1.6 (1.4 - 1.8) | <0.001 | 1997 - 2004 | -0.7 (-1 - -0.4) | <0.001 | 2004 - 2011 | 0.5 (0.2 - 0.8) | 0.002 | 2011 - 2019 | -1.5 (-1.7 - -1.3) | <0.001 | -0.1 (-0.2 - 0) |
| **Russia** | 1990 - 1993 | -0.3 (-4.5 - 4.1) | 0.897 | 1993 - 2003 | -4 (-4.7 - -3.2) | <0.001 | 2003 - 2011 | 0.7 (-0.5 - 1.8) | 0.242 | 2011 - 2019 | -3.9 (-4.8 - -3) | <0.001 | -2.3 (-2.9 - -1.7) |
| **USA** | 1990 - 1997 | 0.6 (0.2 - 0.9) | 0.002 | 1997 - 2005 | -2.1 (-2.5 - -1.8) | <0.001 | 2005 - 2017 | -6 (-6.2 - -5.9) | <0.001 | 2017 - 2019 | -1 (-3.5 - 1.5) | 0.398 | -3.1 (-3.3 - -2.9) |
| **Mexico** | 1990 - 1997 | -1.1 (-1.6 - -0.7) | <0.001 | 1997 - 2007 | -4.4 (-4.7 - -4.1) | <0.001 | 2007 - 2019 | -1.6 (-1.8 - -1.4) | <0.001 |  |  |  | -2.5 (-2.7 - -2.3) |
| **Brazil** | 1990 - 1992 | -2.6 (-4 - -1.1) | 0.001 | 1992 - 2011 | -0.5 (-0.6 - -0.5) | <0.001 | 2011 - 2014 | -4.6 (-6 - -3.2) | <0.001 | 2014 - 2019 | -1.9 (-2.2 - -1.6) | <0.001 | -1.3 (-1.5 - -1.2) |
| **Bangladesh** | 1990 - 2002 | 0.7 (0.4 - 0.9) | <0.001 | 2002 - 2013 | 0.2 (-0.1 - 0.5) | 0.186 | 2013 - 2019 | 2 (1.3 - 2.7) | <0.001 |  |  |  | 0.8 (0.6 - 1) |
| **India** | 1990 - 1992 | -1.7 (-7 - 3.8) | 0.511 | 1992 - 1998 | 2.2 (0.9 - 3.5) | 0.002 | 1998 - 2010 | 0.1 (-0.3 - 0.5) | 0.575 | 2010 - 2019 | 3.3 (2.7 - 3.8) | <0.001 | 1.4 (0.9 - 1.9) |
| **Pakistan** | 1990 - 1994 | 2.2 (1.6 - 2.7) | <0.001 | 1994 - 2001 | 3.2 (2.9 - 3.5) | <0.001 | 2001 - 2014 | 1.1 (1 - 1.2) | <0.001 | 2014 - 2019 | 0.3 (-0.1 - 0.7) | 0.123 | 1.6 (1.5 - 1.8) |
| **Nigeria** | 1990 - 2001 | -0.1 (-0.2 - 0) | 0.094 | 2001 - 2009 | 2.5 (2.3 - 2.8) | <0.001 | 2009 - 2013 | 3.9 (3.1 - 4.8) | <0.001 | 2013 - 2019 | 0.6 (0.3 - 0.9) | <0.001 | 1.3 (1.2 - 1.5) |
| **Tobacco** | | | | | | | | | | | | | |
| **Global** | 1990 - 2002 | 0.9 (0.8 - 0.9) | <0.001 | 2002 - 2009 | -0.2 (-0.4 - 0) | 0.038 | 2009 - 2017 | -1.1 (-1.3 - -1) | <0.001 | 2017 - 2019 | 0.5 (-0.7 - 1.6) | 0.412 | 0 (-0.1 - 0.1) |
| **China** | 1990 - 1998 | 2.6 (2.2 - 2.9) | <0.001 | 1998 - 2004 | 4.1 (3.5 - 4.8) | <0.001 | 2004 - 2016 | -1 (-1.2 - -0.8) | <0.001 | 2016 - 2019 | 1.7 (0.2 - 3.1) | 0.029 | 1.3 (1.1 - 1.5) |
| **Indonesia** | 1990 - 1998 | 3.7 (3.5 - 3.8) | <0.001 | 1998 - 2004 | 1 (0.8 - 1.2) | <0.001 | 2004 - 2013 | 2.3 (2.2 - 2.4) | <0.001 | 2013 - 2019 | 1.5 (1.4 - 1.7) | <0.001 | 2.3 (2.2 - 2.3) |
| **Russia** | 1990 - 1994 | 1.7 (0.3 - 3.2) | 0.019 | 1994 - 1998 | -5.6 (-7.7 - -3.5) | <0.001 | 1998 - 2006 | 0.3 (-0.3 - 0.9) | 0.284 | 2006 - 2019 | 2.8 (2.5 - 3) | <0.001 | 0.8 (0.4 - 1.2) |
| **USA** | 1990 - 1999 | 1 (0.8 - 1.2) | <0.001 | 1999 - 2008 | -1 (-1.3 - -0.8) | <0.001 | 2008 - 2017 | -2.2 (-2.4 - -1.9) | <0.001 | 2017 - 2019 | 0.7 (-1.5 - 2.9) | 0.512 | -0.6 (-0.8 - -0.5) |
| **Mexico** | 1990 - 1996 | -1.7 (-2.1 - -1.3) | <0.001 | 1996 - 2017 | -3.6 (-3.7 - -3.5) | <0.001 | 2017 - 2019 | -1.3 (-3.7 - 1.1) | 0.265 |  |  |  | -3.1 (-3.2 - -2.9) |
| **Brazil** | 1990 - 2000 | 0.8 (0.7 - 1) | <0.001 | 2000 - 2012 | 0 (-0.1 - 0.1) | 0.443 | 2012 - 2016 | -0.6 (-1.2 - 0.1) | 0.097 | 2016 - 2019 | -2.2 (-2.9 - -1.6) | <0.001 | 0 (-0.1 - 0.1) |
| **Bangladesh** | 1990 - 2002 | 0.4 (0.2 - 0.6) | 0.003 | 2002 - 2013 | -0.7 (-1 - -0.4) | <0.001 | 2013 - 2019 | 2 (1.3 - 2.7) | <0.001 |  |  |  | 0.3 (0.1 - 0.5) |
| **India** | 1990 - 1998 | 2 (1.4 - 2.6) | <0.001 | 1998 - 2011 | -0.2 (-0.5 - 0.1) | 0.272 | 2011 - 2015 | 5.4 (2.7 - 8.2) | <0.001 | 2015 - 2019 | 2.3 (0.7 - 4) | 0.009 | 1.5 (1.1 - 2) |
| **Pakistan** | 1990 - 1993 | 2 (0.7 - 3.3) | 0.003 | 1993 - 2002 | 3.5 (3.2 - 3.8) | <0.001 | 2002 - 2010 | 1.3 (1 - 1.7) | <0.001 | 2010 - 2019 | -0.9 (-1.1 - -0.7) | <0.001 | 1.4 (1.2 - 1.5) |
| **Nigeria** | 1990 - 2008 | 0.2 (0.1 - 0.4) | <0.001 | 2008 - 2013 | 2.7 (1.6 - 3.8) | <0.001 | 2013 - 2019 | -0.2 (-0.7 - 0.4) | 0.529 |  |  |  | 0.6 (0.4 - 0.8) |
| **Occupational exposure to asbestos** | | | | | | | | | | | | | |
| **Global** | 1990 -2006 | 0.5 (0.4 - 0.6) | <0.001 | 2006 -2011 | 1 (0.2 - 1.9) | 0.018 | 2011 -2019 | -1.3 (-1.6 - -1) | <0.001 |  |  |  | 0.1 (-0.1 - 0.3) |
| **China** | 1990 -1998 | -1 (-1.7 - -0.4) | 0.004 | 1998 -2011 | 2.9 (2.5 - 3.3) | <0.001 | 2011 -2016 | -3.3 (-5.1 - -1.5) | 0.001 | 2016 -2019 | 1.5 (-1.5 - 4.6) | 0.317 | 0.6 (0.1 - 1.1) |
| **Indonesia** | 1990 -1997 | 5.1 (3.9 - 6.4) | <0.001 | 1997 -2019 | 2 (1.8 - 2.2) | <0.001 |  |  |  |  |  |  | 2.7 (2.4 - 3) |
| **Russia** | 1990 -1994 | 5.4 (2.6 - 8.3) | 0.001 | 1994 -1997 | -7.7 (-15.3 - 0.5) | 0.063 | 1997 -2019 | 0.1 (-0.1 - 0.3) | 0.216 |  |  |  | 0 (-0.9 - 0.9) |
| **USA** | 1990 -2000 | 2.6 (2.3 - 2.9) | <0.001 | 2000 -2012 | -0.4 (-0.6 - -0.2) | 0.002 | 2012 -2017 | -2.5 (-3.5 - -1.4) | <0.001 | 2017 -2019 | 1 (-2.2 - 4.4) | 0.516 | 0.4 (0.1 - 0.7) |
| **Mexico** | 1990 -1996 | -0.7 (-2 - 0.6) | 0.258 | 1996 -2001 | -4 (-6.3 - -1.6) | 0.002 | 2001 -2005 | 0.9 (-3 - 4.8) | 0.649 | 2005 -2019 | -1.1 (-1.5 - -0.7) | <0.001 | -1.3 (-2 - -0.6) |
| **Brazil** | 1990 -1999 | -0.2 (-0.5 - 0.1) | 0.113 | 1999 -2008 | 0.9 (0.5 - 1.2) | <0.001 | 2008 -2015 | 1.6 (1.1 - 2.1) | <0.001 | 2015 -2019 | -0.2 (-1.1 - 0.8) | 0.669 | 0.6 (0.3 - 0.8) |
| **Bangladesh** | 1990 -1996 | -2 (-4.4 - 0.5) | 0.114 | 1996 -2001 | 8.3 (3.3 - 13.5) | 0.002 | 2001 -2012 | -2.1 (-3.2 - -1) | 0.001 | 2012 -2019 | 3 (1 - 5.1) | 0.005 | 0.9 (-0.2 - 2) |
| **India** | 1990 -2007 | 1.6 (1 - 2.2) | <0.001 | 2007 -2010 | -7.8 (-22.3 - 9.3) | 0.331 | 2010 -2019 | 4.9 (3.3 - 6.6) | <0.001 |  |  |  | 1.6 (-0.2 - 3.4) |
| **Pakistan** | 1990 -2002 | 4.9 (4.3 - 5.5) | <0.001 | 2002 -2019 | 1.2 (0.8 - 1.5) | <0.001 |  |  |  |  |  |  | 2.7 (2.4 - 3) |
| **Nigeria** | 1990 -2001 | 0 (-0.9 - 1) | 0.968 | 2001 -2019 | 3.1 (2.7 - 3.6) | <0.001 |  |  |  |  |  |  | 1.9 (1.5 - 2.4) |
| **Ambient Particulate Matter Pollution** | | | | | | | | | | | | | |
| **Global** | 1990 -2009 | 2.1 (2 - 2.1) | <0.001 | 2009 -2014 | 0.9 (0.3 - 1.4) | 0.004 | 2014 -2017 | -0.7 (-2.4 - 1.1) | 0.437 | 2017 -2019 | 3.3 (1.5 - 5.1) | 0.001 | 1.7 (1.4 - 1.9) |
| **China** | 1990 -1995 | 4.4 (3.7 - 5.2) | <0.001 | 1995 -2003 | 6.6 (6.1 - 7) | <0.001 | 2003 -2010 | 3.6 (3 - 4.1) | <0.001 | 2010 -2019 | 1 (0.7 - 1.2) | <0.001 | 3.7 (3.5 - 3.9) |
| **Indonesia** | 1990 -1997 | 7 (6.3 - 7.8) | <0.001 | 1997 -2005 | 1.4 (0.7 - 2.1) | 0.001 | 2005 -2009 | 4.3 (1.6 - 7.1) | 0.003 | 2009 -2019 | 1.6 (1.2 - 2) | <0.001 | 3.2 (2.7 - 3.6) |
| **Russia** | 1990 -1993 | 0.6 (-4.1 - 5.6) | 0.792 | 1993 -2002 | -4.1 (-5.1 - -3.1) | <0.001 | 2002 -2011 | 0.9 (-0.1 - 2) | 0.088 | 2011 -2019 | -3.7 (-4.7 - -2.7) | <0.001 | -2 (-2.7 - -1.3) |
| **USA** | 1990 -1997 | 0.6 (0.2 - 0.9) | 0.004 | 1997 -2005 | -2.1 (-2.5 - -1.8) | <0.001 | 2005 -2017 | -6.1 (-6.2 - -5.9) | <0.001 | 2017 -2019 | -0.2 (-2.9 - 2.6) | 0.892 | -3 (-3.2 - -2.8) |
| **Mexico** | 1990 -1998 | 0.7 (0.2 - 1.3) | 0.01 | 1998 -2007 | -4.6 (-5.1 - -4.1) | <0.001 | 2007 -2019 | -0.5 (-0.8 - -0.3) | 0.001 |  |  |  | -1.5 (-1.7 - -1.3) |
| **Brazil** | 1990 -2000 | 3.3 (3.1 - 3.4) | <0.001 | 2000 -2010 | 1.8 (1.7 - 2) | <0.001 | 2010 -2014 | -3.1 (-4.1 - -2.1) | <0.001 | 2014 -2019 | -0.2 (-0.6 - 0.3) | 0.455 | 1.3 (1.1 - 1.5) |
| **Bangladesh** | 1990 -2000 | 6.1 (5.3 - 6.9) | <0.001 | 2000 -2007 | 2.5 (0.9 - 4.2) | 0.004 | 2007 -2019 | 8.2 (7.6 - 8.8) | <0.001 |  |  |  | 6.1 (5.6 - 6.6) |
| **India** | 1990 -2011 | 4.6 (4.4 - 4.8) | <0.001 | 2011 -2014 | 12.4 (5.1 - 20.1) | 0.001 | 2014 -2019 | 4 (2.5 - 5.6) | <0.001 |  |  |  | 5.3 (4.5 - 6) |
| **Pakistan** | 1990 -1995 | 3.6 (2.5 - 4.8) | <0.001 | 1995 -2000 | 11.9 (10.2 - 13.7) | <0.001 | 2000 -2014 | 6.5 (6.2 - 6.8) | <0.001 | 2014 -2019 | 4 (2.9 - 5.2) | <0.001 | 6.5 (6.1 - 6.9) |
| **Nigeria** | 1990 -2003 | 3.5 (3.2 - 3.9) | <0.001 | 2003 -2009 | 7.2 (5.7 - 8.8) | <0.001 | 2009 -2013 | 12.7 (9 - 16.4) | <0.001 | 2013 -2019 | 5 (3.9 - 6.2) | <0.001 | 5.8 (5.2 - 6.4) |
| **Household air pollution from solid fuels** | | | | | | | | | | | | | |
| **Global** | 1990 -2005 | -1.4 (-1.5 - -1.3) | <0.001 | 2005 -2013 | -4.4 (-4.7 - -4.2) | <0.001 | 2013 -2019 | -3.1 (-3.5 - -2.8) | <0.001 |  |  |  | -2.6 (-2.7 - -2.5) |
| **China** | 1990 -2005 | -1.9 (-2.1 - -1.8) | <0.001 | 2005 -2015 | -6.6 (-6.9 - -6.3) | <0.001 | 2015 -2019 | -4.7 (-5.8 - -3.6) | <0.001 |  |  |  | -3.9 (-4.1 - -3.7) |
| **Indonesia** | 1990 -1996 | -0.1 (-0.5 - 0.2) | 0.451 | 1996 -2010 | -1.3 (-1.4 - -1.2) | <0.001 | 2010 -2015 | -3 (-3.7 - -2.4) | <0.001 | 2015 -2019 | -4.9 (-5.5 - -4.2) | <0.001 | -1.9 (-2 - -1.7) |
| **Russia** | 1990 -2001 | -5.5 (-7 - -4) | <0.001 | 2001 -2019 | -7.3 (-8 - -6.6) | <0.001 |  |  |  |  |  |  | -6.6 (-7.3 - -6) |
| **USA** | N/A | N/A | N/A | N/A | N/A | N/A | N/A | N/A | N/A | N/A | N/A | N/A | N/A |
| **Mexico** | 1990 -1996 | -4.4 (-4.9 - -3.9) | <0.001 | 1996 -2000 | -6.9 (-8.3 - -5.5) | <0.001 | 2000 -2009 | -3.8 (-4.1 - -3.5) | <0.001 | 2009 -2019 | -4.7 (-4.9 - -4.4) | <0.001 | -4.6 (-4.9 - -4.4) |
| **Brazil** | 1990 -1992 | -5.3 (-7.1 - -3.4) | <0.001 | 1992 -2011 | -3.8 (-3.9 - -3.7) | <0.001 | 2011 -2016 | -5.8 (-6.4 - -5.2) | <0.001 | 2016 -2019 | -6.8 (-7.7 - -5.9) | <0.001 | -4.6 (-4.7 - -4.4) |
| **Bangladesh** | 1990 -2006 | 0 (-0.2 - 0.2) | 0.924 | 2006 -2015 | -1.7 (-2.1 - -1.2) | <0.001 | 2015 -2019 | 0.1 (-1.3 - 1.5) | 0.883 |  |  |  | -0.5 (-0.8 - -0.3) |
| **India** | 1990 -1998 | 0.6 (0 - 1.2) | 0.038 | 1998 -2007 | -1.5 (-2.1 - -1) | <0.001 | 2007 -2011 | -3.6 (-6.2 - -1) | 0.009 | 2011 -2019 | -0.7 (-1.3 - -0.2) | 0.014 | -1 (-1.5 - -0.6) |
| **Pakistan** | 1990 -2001 | 1.9 (1.8 - 2.1) | <0.001 | 2001 -2008 | -0.2 (-0.6 - 0.2) | 0.276 | 2008 -2019 | -2.1 (-2.3 - -2) | <0.001 |  |  |  | -0.1 (-0.3 - 0) |
| **Nigeria** | 1990 -2001 | -0.9 (-1 - -0.7) | <0.001 | 2001 -2006 | 1.6 (0.8 - 2.4) | <0.001 | 2006 -2012 | 0.6 (0 - 1.1) | 0.044 | 2012 -2019 | -2.6 (-2.9 - -2.2) | <0.001 | -0.6 (-0.8 - -0.4) |

APC: Annual Percentage Change; AAPC: Average Annual Percentage Change; ASMR: Age Standardized Mortality Rate; N/A: Not available for the USA due to a small sample size.; USA: United States of America.
