# Supplementary material for: Evolving trends in lung cancer risk factors in the ten most populous countries: an analysis of data from the 2019 Global Burden of Disease Study
Source: eClinicalMedicine. 2025 Jan 9;79:103033. doi: 10.1016/j.eclinm.2024.103033 (PMC11833020; doi:10.1016/j.eclinm.2024.103033)
Supplement: Supplementary Tables [file mmc2.docx]

**Caption for Supplementary Figure 1:**

Overall trends in TBL cancer ASMR (Age Standardized Mortality Rate) and Annual Percent Change (APC) by risk factors globally and in the top 10 most populated countries (1990–2019): (A) Tobacco use, (B) Air Pollution, (C) Ambient Particulate Matter Pollution, (D) Household Air Pollution Due to Solid Fuels, and (E) Occupational Exposure to Asbestos. Each panel illustrates the ASMR across years and their APC trends for global and country-specific data, marked by Joinpoints indicating periods of significant change in trends. The legends provide APC values for the respective trends, their associated Confidence Interval and p values.

**Caption for Supplementary Table 1:**

This table presents the results of joinpoint regression analysis for age-standardized TBL cancer mortality rates (ASMR) for both sexes combined, across the years 1990–2019, based on key risk factors: A. Air Pollution, B. Tobacco, C. Occupational Exposure to Asbestos, D. Ambient Particulate Matter Pollution and, E. Household Air Pollution from Solid Fuels.

**Caption for Supplementary Table 2:** Joinpoint Analysis of TBL cancer Mortality for Males (1990–2019) by Risk Factors. This table provides joinpoint analysis of TBLC ASMR trends for males, categorized by the same risk factors as in Supplementary Table 1.

**Caption for Supplementary Table 3:** Joinpoint Analysis of TBL cancer Mortality for Females (1990–2019) by Risk Factors. This table provides joinpoint analysis of TBLC ASMR trends for females, highlighting sex-specific differences in TBLC mortality trends by risk factors.

The analysis in all three tables includes annual percentage change (APC), average annual percentage change (AAPC), and 95% confidence intervals (CI) for each trend segment identified by the joinpoint analysis. Results are stratified globally and by countries with details on observed shifts in trends over the study period. Significant p-values indicate periods of statistically significant changes in TBLC mortality trends.
